# Supplementary material for: Emerging Role of ODC1 in Neurodevelopmental Disorders and Brain Development
Source: Genes (Basel). 2021 Mar 25;12(4):470. doi: 10.3390/genes12040470 (PMC8064465; doi:10.3390/genes12040470)
Supplement: Supplementary file 1 [file genes-12-00470-s001.pdf]

## Supplemental File

## Table S1 Mouse *ODC1* knockout phenotypes from the IMPC (https://www.mousephenotype.org/data/genes/MGI:97402).

| Phenotype | Zygosity | Sex | Procedure | Parameter | Phenotyping Center | Source | P Value |
|---|---|---|---|---|---|---|---|
| preweaning lethality, complete penetrance | homozygote | female | Viability Primary Screen | Viability Outcome | JAX | IMPC | 0.0 |
| preweaning lethality, complete penetrance | homozygote | male | Viability Primary Screen | Viability Outcome | JAX | IMPC | 0.0 |
| decreased lean body mass | heterozygote | female | Body Composition (DEXA lean/fat) | Lean mass | JAX | IMPC | 4.21E-6 |
| decreased lean body mass | heterozygote | male | Body Composition (DEXA lean/fat) | Lean mass | JAX | IMPC | 4.21E-6 |
| decreased total body fat amount | heterozygote | male | Body Composition (DEXA lean/fat) | Fat mass | JAX | IMPC | 3.08E-5 |

## Table S2 Geno2MP Missense SNPs for *ODC1*.

| Variant | AA | Var | # | HPO profiles | heterozygous | homozygous | Phenotypes | polyphen2 | Provean | SIFT | Impact Total |
|---|---|---|---|---|---|---|---|---|---|---|---|
| G84R | G | R | 84 | 72 | 121 | 1 | Nervous System | probably-damaging | Deleterious | Damaging | 125.8 |
| C202R | C | R | 202 | 1 | 1 | 0 | Abnormality of hindbrain morphology | possibly-damaging | Deleterious | Damaging | 103.5 |
| S195G | S | G | 195 | 5 | 6 | 0 | head and neck | possibly-damaging | Deleterious | Damaging | 100.5 |
| T285M | T | M | 285 | 1 | 1 | 0 | cardiovascular | possibly-damaging | Deleterious | Damaging | 85.4 |
| F383L | F | L | 383 | 1 | 1 | 0 | Abnormality of brain morphology | benign | Deleterious | Damaging | 72.9 |
| A258V | A | V | 258 | 4 | 4 | 0 | head and neck | possibly-damaging | Deleterious | Damaging | 72.6 |
| N398S | N | S | 398 | 1 | 1 | 0 | Abnormality of adipose tissue | probably-damaging | Deleterious | Tolerated | 70.35 |
| S410L | S | L | 410 | 1 | 1 | 0 | Abnormal cellular physiology | benign | Deleterious | Damaging | 61.25 |
| D204N | D | N | 204 | 2 | 2 | 0 | nervous system; metabolism/homeostasis | benign | Deleterious | Tolerated | 60.5 |
| D243H | D | H | 243 | 1 | 1 | 0 | Nephrotic syndrome | benign | Deleterious | Damaging | 60.075 |
| V322I | V | I | 322 | 1 | 1 | 0 | skeletal system | possibly-damaging | Neutral | Tolerated | 54.2 |
| R183W | R | W | 183 | 15 | 17 | 0 | nervous system | benign | Deleterious | Damaging | 52.65 |
| G268R | G | R | 268 | 19 | 24 | 0 | nervous system | benign | Deleterious | Damaging | 50 |
| Y122N | Y | N | 122 | 1 | 1 | 0 | Aplasia/Hypoplasia affecting the eye | benign | Deleterious | Damaging | 46.575 |
| R365L | R | L | 365 | 1 | 1 | 0 | Omphalocele | benign | Deleterious | Tolerated | 44.91 |
| A287G | A | G | 287 | 1 | 1 | 0 | Abnormality of brain morphology | benign | Deleterious | Damaging | 44.685 |
| L363F | L | F | 363 | 1 | 1 | 0 | Arnold-Chiari malformation | benign | Deleterious | Tolerated | 39.625 |
| R178K | R | K | 178 | 7 | 6 | 1 | nervous system | benign | Neutral | Tolerated | 31.25 |
| D15N | D | N | 15 | 1 | 1 | 0 | Retinitis pigmentosa | benign | Neutral | Damaging | 26.75 |
| V62I | V | I | 62 | 1 | 1 | 0 | Abnormality of the musculature | benign | Neutral | Damaging | 23.6875 |
| T157A | T | A | 157 | 1 | 1 | 0 | Dystonia | benign | Deleterious | Tolerated | 22.225 |
| A452V | A | V | 452 | 1 | 1 | 0 | Abnormality of the urinary system | benign | Neutral | Damaging | 22.15 |
| S456L | S | L | 456 | 2 | 3 | 0 | Intellectual disability | benign | Neutral | Tolerated | 11.65 |
| V254I | V | I | 254 | 2 | 2 | 0 | cardiovascular and nervous | benign | Neutral | Tolerated | 10.95 |
| H335Y | H | Y | 335 | 1 | 1 | 0 | Seizures | benign | Neutral | Tolerated | 5.346 |
| A452T | A | T | 452 | 4 | 4 | 0 | Renal agenesis | benign | Neutral | Tolerated | 4.87 |
| H376Y | H | Y | 376 | 1 | 2 | 0 | Abnormality of brain morphology | benign | Neutral | Tolerated | 4.7625 |
| T301R | T | R | 301 | 1 | 1 | 0 | Seizures | benign | Neutral | Tolerated | 2.00025 |
| T301M | T | M | 301 | 1 | 1 | 0 | Agenesis of corpus callosum | benign | Neutral | Tolerated | 1.871 |
| F4S | F | S | 4 | 1 | 1 | 0 | Bicuspid aortic valve | benign | Neutral | Tolerated | 0 |
| I23L | I | L | 23 | 3 | 3 | 0 | Abnormality of the lung | benign | Neutral | Tolerated | 0 |
| S32F | S | F | 32 | 1 | 1 | 0 | Abnormality of blood and blood-forming tissues | benign | Neutral | Tolerated | 0 |
| S100R | S | R | 100 | 1 | 1 | 0 | Abnormality of the abdominal wall | benign | Neutral | Tolerated | 0 |
| Q129R | Q | R | 129 | 2 | 2 | 0 | Intellectual disability | benign | Neutral | Tolerated | 0 |
| A143V | A | V | 143 | 4 | 4 | 0 | lissencephaly | benign | Neutral | Tolerated | 0 |
| V163I | V | I | 163 | 1 | 2 | 0 | Seizures | benign | Neutral | Tolerated | 0 |
| N188S | N | S | 188 | 1 | 1 | 0 | Abnormal hemoglobin | benign | Neutral | Tolerated | 0 |
| V225A | V | A | 225 | 1 | 1 | 0 | Absence of the sacrum | benign | Neutral | Tolerated | 0 |
| G253R | G | R | 253 | 11 | 14 | 0 | musculature | benign | Neutral | Tolerated | 0 |
| G253S | G | S | 253 | 6 | 6 | 0 | cardiovascular | benign | Neutral | Tolerated | 0 |
| D307G | D | G | 307 | 1 | 1 | 0 | Abnormality of the lung | benign | Neutral | Tolerated | 0 |
| S309L | S | L | 309 | 1 | 1 | 0 | Global developmental delay | benign | Neutral | Tolerated | 0 |
| S310N | S | N | 310 | 1 | 1 | 0 | Intellectual disability | benign | Neutral | Tolerated | 0 |
| K349Q | K | Q | 349 | 3 | 3 | 0 | Abnormal eye morphology | benign | Neutral | Tolerated | 0 |
| R369C | R | C | 369 | 2 | 2 | 0 | Nephronophthisis | benign | Neutral | Tolerated | 0 |
| R449C | R | C | 449 | 1 | 1 | 0 | cardiovascular system | benign | Neutral | Tolerated | 0 |
| S458G | S | G | 458 | 2 | 2 | 0 | nervous system | benign | Neutral | Tolerated | 0 |

# Table S3 Allele frequency for ODC G84R (rs138359527) from population genomic projects.

| Study | Sub Study | Population | Sub Population | Sub Population | C Freq | C Count | T Freq | T Count |
|---|---|---|---|---|---|---|---|---|
| 1000GENOMES | phase_3 | SAS | GIH | Gujarati Indians in Houston, TX | 0.985 | 203 | 0.015 | 3 |
| 1000GENOMES | phase_3 | SAS | PJL | Punjabi in Lahore, Pakistan | 0.99 | 190 | 0.01 | 2 |
| gnomADe | exome | sas | | | 0.992 | 30257 | 0.008 | 233 |
| 1000GENOMES | phase_3 | AMR | PUR | Puerto Ricans from Puerto Rico | 0.995 | 207 | 0.005 | 1 |
| 1000GENOMES | phase_3 | EUR | FIN | Finnish in Finland | 0.995 | 197 | 0.005 | 1 |
| gnomADg | genome | sas | | | 0.995 | 3020 | 0.005 | 16 |
| UK10K | | TWINSUK | | | 0.996 | 3560 | 0.004 | 14 |
| gnomADe | exome | nfe | | | 0.997 | 112938 | 0.003 | 314 |
| gnomADg | genome | nfe | | | 0.997 | 64359 | 0.003 | 211 |
| UK10K | | ALSPAC | | | 0.997 | 3841 | 0.003 | 13 |
| gnomADe | exome | oth | | | 0.998 | 6104 | 0.002 | 14 |
| gnomADe | exome | amr | | | 0.999 | 34444 | 0.001 | 20 |
| gnomADg | genome | ami | | | 0.999 | 899 | 0.001 | 1 |
| gnomADg | genome | amr | | | 0.999 | 13641 | 0.001 | 13 |
| gnomADg | genome | asj | | | 0.999 | 3320 | 0.001 | 2 |
| gnomADg | genome | oth | | | 0.999 | 2149 | 0.001 | 3 |
| gnomADe | exome | asj | | | 0.9995003 | 10001 | 0.0004997 | 5 |
| gnomADg | genome | afr | | | 0.999571796 | 42018 | 0.000428204 | 18 |
| gnomADg | genome | fin | | | 0.999617883 | 10464 | 0.000382117 | 4 |
| gnomADe | exome | fin | | | 0.999722171 | 21590 | 0.000277829 | 6 |
| gnomADe | exome | afr | | | 0.999753755 | 16240 | 0.000246245 | 4 |
| gnomADe | exome | eas | | | 0.999836815 | 18381 | 0.000163185 | 3 |
| gnomADg | genome | eas | | | 1 | 3134 | 0 | 0 |
| 1000GENOMES | phase_3 | AFR | ACB | African Caribbeans in Barbados | 1 | 192 | 0 | 0 |
| 1000GENOMES | phase_3 | AFR | ASW | Americans of African Ancestry in SW USA | 1 | 122 | 0 | 0 |
| 1000GENOMES | phase_3 | AFR | ESN | Esan in Nigeria | 1 | 198 | 0 | 0 |
| 1000GENOMES | phase_3 | AFR | GWD | Gambian in Western Divisions in the Gambia | 1 | 226 | 0 | 0 |
| 1000GENOMES | phase_3 | AFR | LWK | Luhya in Webuye, Kenya | 1 | 198 | 0 | 0 |
| 1000GENOMES | phase_3 | AFR | MSL | Mende in Sierra Leone | 1 | 170 | 0 | 0 |
| 1000GENOMES | phase_3 | AFR | YRI | Yoruba in Ibadan, Nigeria | 1 | 216 | 0 | 0 |
| 1000GENOMES | phase_3 | AMR | CLM | Colombians from Medellin, Colombia | 1 | 188 | 0 | 0 |
| 1000GENOMES | phase_3 | AMR | MXL | Mexican Ancestry from Los Angeles USA | 1 | 128 | 0 | 0 |
| 1000GENOMES | phase_3 | AMR | PEL | Peruvians from Lima, Peru | 1 | 170 | 0 | 0 |
| 1000GENOMES | phase_3 | EAS | CDX | Chinese Dai in Xishuangbanna, China | 1 | 186 | 0 | 0 |
| 1000GENOMES | phase_3 | EAS | CHB | Han Chinese in Beijing, China | 1 | 206 | 0 | 0 |
| 1000GENOMES | phase_3 | EAS | CHS | Southern Han Chinese | 1 | 210 | 0 | 0 |
| 1000GENOMES | phase_3 | EAS | JPT | Japanese in Tokyo, Japan | 1 | 208 | 0 | 0 |
| 1000GENOMES | phase_3 | EAS | KHV | Kinh in Ho Chi Minh City, Vietnam | 1 | 198 | 0 | 0 |
| 1000GENOMES | phase_3 | EUR | CEU | Utah Residents (CEPH) with Northern and Western European Ancestry | 1 | 198 | 0 | 0 |
| 1000GENOMES | phase_3 | EUR | GBR | British in England and Scotland | 1 | 182 | 0 | 0 |
| 1000GENOMES | phase_3 | EUR | IBS | Iberian Population in Spain | 1 | 214 | 0 | 0 |
| 1000GENOMES | phase_3 | EUR | TSI | Toscani in Italia | 1 | 214 | 0 | 0 |
| 1000GENOMES | phase_3 | SAS | BEB | Bengali from Bangladesh | 1 | 172 | 0 | 0 |
| 1000GENOMES | phase_3 | SAS | ITU | Indian Telugu from the UK | 1 | 204 | 0 | 0 |
| 1000GENOMES | phase_3 | SAS | STU | Sri Lankan Tamil from the UK | 1 | 204 | 0 | 0 |

## Table S4 PheWAS for ODC G84R (rs138359527) extracted from Open Targets Genetics.

| Trait | P-value | Beta | Odds Ratio | Author (Year) |
|---|---|---|---|---|
| Hiatus hernia \| non-cancer illness code, self-reported | 0.000611209 | 0.552269905 | 1.737191806 | UKB Neale v2 |
| Cetirizine \| treatment/medication code | 0.00104885 | 0.753210099 | 2.123806716 | UKB Neale v2 |
| Spine arthritis/spondylitis \| non-cancer illness code, self-reported | 0.00210866 | 0.801101223 | 2.227993096 | UKB Neale v2 |
| Fracture of ankle and foot | 0.00623 | 0.821 | 2.272771473 | UKB SAIGE |
| Mean signal-to-noise ratio (snr), (left) | 0.0081056 | -718.818 | | UKB Neale v2 |
| Felt very upset when reminded of stressful experience in past month | 0.00857595 | 0.0916764 | | UKB Neale v2 |
| Urethral stricture (not specified as infectious) | 0.009 | -0.651 | 0.521523992 | UKB SAIGE |
| Reaction time | 0.00909346 | 0.120451 | | Davies G |
| Nervous feelings | 0.00915901 | -0.149873195 | 0.860817126 | UKB Neale v2 |
| Inguinal hernia | 0.00927 | -0.324 | 0.723250242 | UKB SAIGE |

## Table S5 HPO variants for *ODC1* from Geno2MP.

| rsId | Allele Change | gnomAD Allele | gnomAD rank | HPO Count | heterozygous Count | homozygous Count | Annotation |
|---|---|---|---|---|---|---|---|
| rs138359527 | C>T | 0.0023907 | 16 | 72 | 121 | 1 | missense |
| rs28362399 | A>G | 0.00763489 | 9 | 32 | 61 | 0 | intron |
| rs28362396 | T>C | 0.0158858 | 6 | 24 | 38 | 4 | intron |
| rs28362409 | C>T | 0.0016156 | 24 | 23 | 32 | 0 | intron |
| rs115655138 | C>T | 0.00142553 | 26 | 22 | 28 | 0 | intron |
| rs201790435 | G>A | 9.04E-04 | 35 | 21 | 28 | 0 | intron |
| rs192090667 | C>T | 0.0216535 | 1 | 20 | 27 | 4 | intron |
| rs61733046 | C>T | 0.00104276 | 31 | 19 | 24 | 0 | missense |
| rs13396481 | C>T | 0.00185292 | 22 | 18 | 26 | 0 | intron |
| NA | G>A | 0.00106587 | 30 | 18 | 19 | 4 | synonymous |
| rs115102834 | G>A | 1.07E-04 | 72 | 15 | 17 | 0 | missense |
| rs143840340 | G>A | 0.0163755 | 4 | 13 | 20 | 0 | intron |
| rs150152038 | C>G | 2.86E-04 | 52 | 11 | 14 | 0 | missense |
| rs28362381 | T>C | 0.0193076 | 2 | 10 | 15 | 1 | intron |
| NA | A>C | 1.19E-05 | 150 | 9 | 14 | 0 | synonymous |
| rs189662188 | A>G | 0.00142477 | 27 | 8 | 9 | 0 | 3-prime-UTR |
| NA | A>T | 0.00116175 | 29 | 8 | 9 | 0 | intron |
| rs180900746 | A>C | 0.00237835 | 17 | 7 | 7 | 0 | intron |
| rs61733045 | C>T | 5.17E-05 | 94 | 7 | 6 | 1 | missense |
| NA | G>A | 0 | 195 | 7 | 7 | 0 | intron |
| rs7558222 | T>A | 0.0162433 | 5 | 7 | 7 | 0 | intron |
| rs28362411 | T>C | 2.66E-04 | 53 | 7 | 7 | 0 | synonymous |
| NA | A>AC | 0.00309096 | 15 | 6 | 12 | 0 | intron |
| rs183432213 | A>G | 3.30E-04 | 47 | 6 | 7 | 0 | synonymous |
| NA | A>G | 2.93E-04 | 50 | 6 | 7 | 0 | 3-prime-UTR |
| rs150152038 | C>T | 4.21E-05 | 105 | 6 | 6 | 0 | missense |
| rs188227508 | C>T | 3.60E-05 | 110 | 6 | 6 | 0 | synonymous |
| rs28362398 | G>C | 7.08E-04 | 39 | 6 | 6 | 0 | intron |
| NA | C>A | 0 | 202 | 5 | 5 | 0 | intron |
| NA | C>A | 0 | 219 | 5 | 5 | 0 | intron |
| rs192347282 | C>T | 0.0044144 | 13 | 5 | 6 | 0 | intron-near-splice |
| NA | C>T | 1.95E-04 | 56 | 5 | 7 | 0 | synonymous |
| rs144846017 | C>T | 1.39E-04 | 65 | 5 | 6 | 0 | synonymous |
| NA | C>T | 7.47E-05 | 84 | 5 | 8 | 0 | intron |
| rs145483973 | G>A | 0.0020752 | 19 | 5 | 5 | 0 | intron |
| NA | G>A | 9.54E-05 | 73 | 5 | 5 | 0 | intron |
| rs28362372 | GGGC>G | 2.87E-04 | 51 | 5 | 1 | 4 | 5-prime-UTR |
| rs189035164 | T>A | 0 | 223 | 5 | 8 | 0 | intron |
| NA | T>C | 1.19E-05 | 148 | 5 | 6 | 0 | missense-near-splice |

# Table S6 Phenotypes in HPO for Abnormality of the nervous system for ODC G84R (rs138359527).

| #C/T | T/T | Status | HPOcode:broad | HPOterm:medium | HPOcode:medium | HPOterm:narrow | HPOcode:narrow |
|---|---|---|---|---|---|---|---|
| 6 | 0 | affected | HP:0000707; | Neurodevelopmental abnormality; | HP:0012759; | Intellectual disability; | HP:0001249; |
| 4 | 0 | affected | HP:0000707; | Abnormality of nervous system physiology; | HP:0012638; | Seizures; | HP:0001250; |
| 3 | 1 | affected | HP:0000707; | Abnormality of nervous system physiology; | HP:0012638; | Epileptic encephalopathy; | HP:0200134; |
| 3 | 0 | affected | HP:0000707; | Abnormality of brain morphology; | HP:0012443; | Agenesis of corpus callosum; | HP:0001274; |
| 3 | 0 | affected | HP:0000707; | Abnormality of nervous system morphology; | HP:0012639; | | |
| 2 | 0 | affected | HP:0000707; | Morphological abnormality of the central nervous system; | HP:0002011; | Neurodegeneration; | HP:0002180; |
| 2 | 0 | affected | HP:0000707; | Morphological abnormality of the central nervous system; | HP:0002011; | | |
| 2 | 0 | affected | HP:0000707; | Abnormality of brain morphology; | HP:0012443; | Abnormality of hindbrain morphology; | HP:0002438; |
| 2 | 0 | affected | HP:0000707; | Abnormality of brain morphology; | HP:0012443; | | |
| 1 | 0 | affected | HP:0000707;HP:0000707; | Behavioral abnormality;Neurodevelopmental abnormality; | HP:0000708;HP:0012759; | Intellectual disability; | HP:0001249; |
| 1 | 0 | affected | HP:0000707; | Seizures; | HP:0001250; | | |
| 1 | 0 | affected | HP:0000707;HP:0000707; | Abnormality of brain morphology;Abnormality of nervous system physiology; | HP:0012443;HP:0012638; | microcephaly;Seizures; | HP:0000252;HP:0001250; |
| 1 | 0 | affected | HP:0000707;HP:0000152; | Abnormality of brain morphology;Abnormality of skull size; | HP:0012443;HP:0000240; | | |
| 1 | 0 | affected | HP:0000707;HP:0003011;HP:0003011 | Abnormality of nervous system physiology;Abnormal muscle physiology;Abnormal muscle physiology | HP:0012638;HP:0011804;HP:0011804 | Fatigable weakness;Progressive muscle weakness | HP:0003473;HP:0003323 |
| 1 | 0 | affected | HP:0000707; | Abnormality of nervous system morphology; | HP:0012639; | Leukodystrophy; | HP:0002415; |
| 1 | 0 | affected | HP:0000707;HP:0002086;HP:0000707 | Neurodevelopmental abnormality;Functional respiratory abnormality;Abnormality of nervous system physiology | HP:0012759;HP:0002795;HP:0012638 | Global developmental delay;Breathing dysregulation;Babinski sign | HP:0001263;HP:0005957;HP:0003487 |
| 1 | 0 | affected | HP:0000707; | Neurodevelopmental abnormality; | HP:0012759; | Global developmental delay; | HP:0001263; |
| 2 | 0 | relative | HP:0000707;HP:0000707; | Behavioral abnormality;Neurodevelopmental abnormality; | HP:0000708;HP:0012759; | Intellectual disability; | HP:0001249; |
| 2 | 0 | relative | HP:0000707; | Abnormality of brain morphology; | HP:0012443; | Abnormality of hindbrain morphology; | HP:0002438; |
| 2 | 0 | relative | HP:0000707; | Abnormality of nervous system physiology; | HP:0012638; | Seizures; | HP:0001250; |
| 2 | 0 | relative | HP:0000707; | Abnormality of nervous system physiology; | HP:0012638; | Epileptic encephalopathy; | HP:0200134; |
| 2 | 0 | relative | HP:0000707;HP:0003011;HP:0003011 | Abnormality of nervous system physiology;Abnormal muscle physiology;Abnormal muscle physiology | HP:0012638;HP:0011804;HP:0011804 | Fatigable weakness;Progressive muscle weakness | HP:0003473;HP:0003323 |
| 1 | 0 | relative | HP:0000707; | Morphological abnormality of the central nervous system; | HP:0002011; | | |
| 1 | 0 | relative | HP:0000707;HP:0000152; | Abnormality of brain morphology;Abnormality of skull size; | HP:0012443;HP:0000240; | | |
| 1 | 0 | relative | HP:0000707; | Neurodevelopmental abnormality; | HP:0012759; | Intellectual disability; | HP:0001249; |
| 1 | 0 | relative | HP:0000707; | Neurodevelopmental abnormality; | HP:0012759; | Global developmental delay; | HP:0001263; |

# Table S7 Scoring of eQTL SNPs for rs9287719 LD block

| RSID | Score | GWAS | Nerve - Tibial p-value | Nerve Tibial NES | AMR | EAS | SAS | AFR | EUR | CADD | RegulomeDB | ENCODE Hits | ROADMAP Hits | GeneHancer |
|---|---|---|---|---|---|---|---|---|---|---|---|---|---|---|
| rs9287719 | 12.6 | prostate carcinoma | 2.80E-07 | -0.13 | 0.56 | 0.62 | 0.40 | 0.77 | 0.52 | 0.18 | 5 | 21 | 48 | |
| rs7592691 | 727.0 | | 2.80E-07 | -0.13 | 0.56 | 0.62 | 0.40 | 0.78 | 0.52 | 14.54 | 7 | 18 | 28 | eQTLs,C-HiC |
| rs7600587 | 591.8 | | 2.80E-07 | -0.13 | . | . | . | . | . | 9.86 | 4 | 22 | 32 | eQTLs,C-HiC |
| rs7605461 | 575.1 | | 2.80E-07 | -0.13 | 0.56 | 0.62 | 0.40 | 0.77 | 0.52 | 7.01 | 4 | 31 | 39 | eQTLs,C-HiC |
| rs2024431 | 537.7 | | 2.80E-07 | -0.13 | 0.56 | 0.63 | 0.40 | 0.78 | 0.52 | 6.81 | 5 | 23 | 46 | eQTLs,C-HiC |
| rs7600495 | 499.5 | | 2.80E-07 | -0.13 | 0.56 | 0.62 | 0.40 | 0.77 | 0.52 | 9.80 | 5 | 16 | 29 | eQTLs,C-HiC |
| rs6755548 | 497.7 | | 2.80E-07 | -0.13 | 0.56 | 0.62 | 0.40 | 0.77 | 0.52 | 7.66 | 5 | 16 | 44 | eQTLs,C-HiC |
| rs4555317 | 468.3 | | 3.80E-07 | -0.13 | 0.56 | 0.62 | 0.40 | 0.77 | 0.52 | 5.78 | 5 | 17 | 59 | eQTLs,C-HiC |
| rs2010080 | 460.7 | | 2.80E-07 | -0.13 | . | . | . | . | . | 7.31 | 7 | 14 | 45 | eQTLs,C-HiC |
| rs728134 | 404.7 | | 2.80E-07 | -0.13 | 0.56 | 0.63 | 0.40 | 0.77 | 0.52 | 7.58 | 7 | 17 | 31 | eQTLs,C-HiC |
| rs7600728 | 403.1 | | 2.80E-07 | -0.13 | 0.56 | 0.62 | 0.40 | 0.77 | 0.52 | 5.76 | 4 | 24 | 39 | eQTLs,C-HiC |
| rs4233883 | 382.6 | | 4.00E-07 | -0.13 | 0.56 | 0.62 | 0.41 | 0.77 | 0.52 | 6.96 | 4 | 21 | 26 | eQTLs,C-HiC |
| rs55738250 | 372.9 | | 2.80E-07 | -0.13 | 0.56 | 0.62 | 0.40 | 0.78 | 0.52 | 5.33 | #N/A | 21 | 42 | eQTLs,C-HiC |
| rs3900609 | 371.2 | | 1.60E-06 | -0.12 | 0.56 | 0.63 | 0.39 | 0.77 | 0.49 | 9.28 | 7 | 9 | 26 | eQTLs,C-HiC |
| rs10167129 | 358.8 | | 2.80E-07 | -0.13 | 0.56 | 0.62 | 0.40 | 0.78 | 0.52 | 8.97 | 4 | 9 | 24 | eQTLs,C-HiC |
| rs6712406 | 349.9 | | 2.80E-07 | -0.13 | 0.56 | 0.62 | 0.40 | 0.77 | 0.52 | 6.36 | 5 | 16 | 33 | eQTLs,C-HiC |
| rs4555318 | 335.4 | | 2.80E-07 | -0.13 | 0.56 | 0.62 | 0.40 | 0.77 | 0.52 | 4.09 | 5 | 19 | 58 | eQTLs,C-HiC |
| rs10454738 | 333.0 | | 2.80E-07 | -0.13 | 0.56 | 0.62 | 0.40 | 0.77 | 0.52 | 4.69 | 5 | 21 | 45 | eQTLs,C-HiC |
| rs7593496 | 324.0 | | 2.80E-07 | -0.13 | 0.56 | 0.62 | 0.40 | 0.77 | 0.52 | 3.52 | 2b | 32 | 43 | eQTLs,C-HiC |
| rs10929688 | 321.0 | | 2.80E-07 | -0.13 | 0.56 | 0.63 | 0.40 | 0.78 | 0.52 | 7.13 | 5 | 6 | 32 | eQTLs,C-HiC |
| rs7563894 | 310.4 | | 2.80E-07 | -0.13 | 0.57 | 0.62 | 0.40 | 0.77 | 0.52 | 9.13 | 5 | 15 | 14 | eQTLs,C-HiC |
| rs6732671 | 294.5 | | 1.60E-06 | -0.12 | 0.56 | 0.63 | 0.40 | 0.77 | 0.49 | 7.85 | #N/A | 12 | 16 | eQTLs,C-HiC |
| rs11674433 | 287.8 | | 2.80E-07 | -0.13 | 0.56 | 0.63 | 0.40 | 0.78 | 0.52 | 4.57 | 5 | 23 | 35 | eQTLs,C-HiC |
| rs6759100 | 285.5 | | 2.80E-07 | -0.13 | 0.56 | 0.62 | 0.40 | 0.77 | 0.52 | 4.39 | 6 | 17 | 39 | eQTLs,C-HiC |
| rs7600820 | 285.5 | | 2.80E-07 | -0.13 | 0.56 | 0.62 | 0.40 | 0.77 | 0.52 | 4.08 | 4 | 25 | 39 | eQTLs,C-HiC |
| rs12692415 | 282.9 | | 2.80E-07 | -0.13 | 0.56 | 0.62 | 0.40 | 0.77 | 0.52 | 6.74 | 6 | 12 | 25 | eQTLs,C-HiC |
| rs4613248 | 282.2 | | 2.80E-07 | -0.13 | 0.56 | 0.62 | 0.40 | 0.77 | 0.52 | 4.03 | 7 | 19 | 46 | eQTLs,C-HiC |
| rs4629131 | 273.2 | | 2.80E-07 | -0.13 | 0.56 | 0.62 | 0.40 | 0.77 | 0.52 | 3.55 | 7 | 20 | 52 | eQTLs,C-HiC |
| rs6727884 | 272.6 | | 2.80E-07 | -0.13 | 0.56 | 0.62 | 0.40 | 0.78 | 0.52 | 4.19 | 5 | 18 | 42 | eQTLs,C-HiC |
| rs7601748 | 268.3 | | 3.90E-06 | -0.12 | 0.55 | 0.63 | 0.39 | 0.77 | 0.49 | 10.32 | 7 | 5 | 16 | eQTLs,C-HiC |
| rs715247 | 265.8 | | 2.80E-07 | -0.13 | 0.56 | 0.62 | 0.40 | 0.77 | 0.52 | 6.48 | 5 | 8 | 26 | eQTLs,C-HiC |
| rs7600695 | 254.2 | | 2.80E-07 | -0.13 | 0.56 | 0.62 | 0.40 | 0.77 | 0.52 | 3.63 | 3a | 23 | 39 | eQTLs,C-HiC |
| rs6706542 | 251.5 | | 2.80E-07 | -0.13 | 0.56 | 0.62 | 0.40 | 0.77 | 0.52 | 3.40 | 4 | 24 | 45 | eQTLs,C-HiC |
| rs7582775 | 248.1 | | 2.80E-07 | -0.13 | 0.56 | 0.62 | 0.40 | 0.77 | 0.52 | 2.73 | 4 | 32 | 52 | eQTLs,C-HiC |
| rs759402 | 242.7 | | 2.80E-07 | -0.13 | . | . | . | . | . | 3.07 | 6 | 21 | 50 | eQTLs,C-HiC |
| rs6733416 | 228.6 | | 2.80E-07 | -0.13 | 0.56 | 0.62 | 0.40 | 0.77 | 0.52 | 2.89 | 3a | 27 | 44 | eQTLs,C-HiC |
| rs6751398 | 224.2 | | 2.80E-07 | -0.13 | 0.56 | 0.62 | 0.40 | 0.77 | 0.52 | 4.87 | 6 | 18 | 23 | eQTLs,C-HiC |
| rs2884233 | 220.6 | | 2.80E-07 | -0.13 | 0.56 | 0.62 | 0.40 | 0.77 | 0.52 | 4.01 | 5 | 18 | 32 | eQTLs,C-HiC |
| rs12464908 | 219.2 | | 2.80E-07 | -0.13 | 0.56 | 0.63 | 0.40 | 0.77 | 0.52 | 3.22 | 7 | 13 | 50 | eQTLs,C-HiC |
| rs12622765 | 212.0 | | 2.80E-07 | -0.13 | 0.56 | 0.63 | 0.40 | 0.78 | 0.52 | 4.42 | 6 | 17 | 26 | eQTLs,C-HiC |
| rs6735074 | 207.1 | | 2.80E-07 | -0.13 | 0.56 | 0.63 | 0.40 | 0.77 | 0.52 | 3.98 | 2c | 10 | 34 | eQTLs,C-HiC |
| rs6727768 | 203.2 | | 2.80E-07 | -0.13 | 0.56 | 0.62 | 0.40 | 0.77 | 0.52 | 2.57 | 5 | 21 | 53 | eQTLs,C-HiC |
| rs6759714 | 199.6 | | 2.80E-07 | -0.13 | 0.56 | 0.62 | 0.40 | 0.77 | 0.52 | 7.13 | 7 | 7 | 16 | eQTLs,C-HiC |
| rs13034017 | 198.1 | | 2.80E-07 | -0.13 | 0.56 | 0.62 | 0.40 | 0.77 | 0.52 | 4.50 | 6 | 14 | 24 | eQTLs,C-HiC |
| rs6710033 | 195.9 | | 2.80E-07 | -0.13 | 0.56 | 0.62 | 0.40 | 0.77 | 0.52 | 4.08 | 4 | 16 | 27 | eQTLs,C-HiC |
| rs6432113 | 189.3 | | 2.80E-07 | -0.13 | 0.56 | 0.62 | 0.40 | 0.77 | 0.52 | 3.79 | 4 | 15 | 29 | eQTLs,C-HiC |
| rs12692418 | 188.1 | | 2.80E-07 | -0.13 | 0.56 | 0.62 | 0.40 | 0.77 | 0.52 | 4.18 | 6 | 15 | 24 | eQTLs,C-HiC |
| rs11687023 | 184.7 | | 2.80E-07 | -0.13 | 0.56 | 0.62 | 0.40 | 0.77 | 0.52 | 3.62 | 7 | 6 | 39 | eQTLs,C-HiC |
| rs4997811 | 184.5 | | 2.80E-07 | -0.13 | 0.56 | 0.62 | 0.41 | 0.78 | 0.52 | 3.54 | #N/A | 20 | 25 | eQTLs,C-HiC |
| rs963831 | 184.2 | | 2.80E-07 | -0.13 | 0.56 | 0.63 | 0.40 | 0.77 | 0.52 | 3.49 | 7 | 17 | 30 | eQTLs,C-HiC |
| rs6432112 | 182.5 | | 2.80E-07 | -0.13 | 0.56 | 0.62 | 0.40 | 0.77 | 0.52 | 3.58 | 4 | 15 | 29 | eQTLs,C-HiC |
| rs1541573 | 182.0 | | 3.10E-07 | -0.13 | 0.49 | 0.60 | 0.40 | 0.78 | 0.52 | 3.64 | 7 | 15 | 30 | eQTLs,C-HiC |
| rs6725354 | 180.1 | | #N/A | #N/A | 0.75 | 0.75 | 0.53 | 0.81 | 0.79 | 3.53 | 5 | 16 | 30 | eQTLs,C-HiC |
| rs6714795 | 177.7 | | 1.60E-06 | -0.12 | 0.56 | 0.63 | 0.40 | 0.77 | 0.49 | 5.23 | 6 | 12 | 16 | eQTLs,C-HiC |
| rs6432110 | 176.5 | | 2.80E-07 | -0.13 | 0.56 | 0.62 | 0.40 | 0.77 | 0.52 | 2.35 | 4 | 32 | 33 | eQTLs,C-HiC |
| rs2010073 | 170.2 | | 2.80E-07 | -0.13 | 0.56 | 0.62 | 0.40 | 0.77 | 0.52 | 3.62 | 5 | 10 | 32 | eQTLs,C-HiC |
| rs58633964 | 165.1 | | #N/A | #N/A | . | . | . | . | . | 4.03 | 5 | 8 | 27 | eQTLs,C-HiC |
| rs4233882 | 150.8 | | 2.80E-07 | -0.13 | 0.56 | 0.62 | 0.41 | 0.78 | 0.52 | 2.79 | 4 | 21 | 26 | eQTLs,C-HiC |
| rs1990612 | 150.3 | | 3.90E-06 | -0.12 | 0.56 | 0.63 | 0.39 | 0.77 | 0.49 | 2.42 | 4 | 11 | 45 | eQTLs,C-HiC |
| rs12467642 | 148.1 | | 2.80E-07 | -0.13 | 0.56 | 0.63 | 0.40 | 0.77 | 0.52 | 2.15 | 6 | 14 | 49 | eQTLs,C-HiC |
| rs6432124 | 147.8 | | 2.80E-07 | -0.13 | 0.56 | 0.62 | 0.41 | 0.77 | 0.52 | 4.00 | 6 | 10 | 22 | eQTLs,C-HiC |
| rs4453662 | 144.9 | | 2.80E-07 | -0.13 | 0.56 | 0.62 | 0.40 | 0.77 | 0.52 | 2.30 | 3a | 18 | 38 | eQTLs,C-HiC |
| rs10195802 | 141.9 | | 2.80E-07 | -0.13 | 0.56 | 0.63 | 0.40 | 0.77 | 0.52 | 2.96 | 5 | 16 | 27 | eQTLs,C-HiC |
| rs4405729 | 136.8 | | 2.80E-07 | -0.13 | 0.56 | 0.62 | 0.40 | 0.77 | 0.52 | 2.74 | 5 | 15 | 29 | eQTLs,C-HiC |
| rs55861579 | 136.7 | | 7.10E-07 | -0.12 | 0.56 | 0.62 | 0.40 | 0.77 | 0.51 | 3.70 | #N/A | 9 | 22 | eQTLs,C-HiC |
| rs12692416 | 133.0 | | 2.80E-07 | -0.13 | 0.56 | 0.62 | 0.40 | 0.77 | 0.52 | 5.32 | 6 | 7 | 13 | eQTLs,C-HiC |
| rs10929686 | 132.6 | | 2.80E-07 | -0.13 | 0.56 | 0.62 | 0.40 | 0.77 | 0.52 | 2.82 | 6 | 14 | 28 | eQTLs,C-HiC |
| rs6432123 | 132.1 | | 2.80E-07 | -0.13 | 0.56 | 0.62 | 0.40 | 0.77 | 0.52 | 3.88 | 6 | 8 | 20 | eQTLs,C-HiC |
| rs10191883 | 131.4 | | 2.80E-07 | -0.13 | 0.56 | 0.62 | 0.40 | 0.77 | 0.52 | 1.71 | 7 | 20 | 52 | eQTLs,C-HiC |
| rs728281 | 127.8 | | 2.80E-07 | -0.13 | 0.56 | 0.62 | 0.40 | 0.77 | 0.52 | 8.52 | 5 | 4 | 6 | eQTLs,C-HiC |
| rs1541574 | 126.8 | | 3.10E-07 | -0.13 | 0.49 | 0.60 | 0.40 | 0.78 | 0.52 | 3.96 | 6 | 8 | 19 | eQTLs,C-HiC |
| rs10201077 | 123.2 | | 2.80E-07 | -0.13 | 0.56 | 0.63 | 0.40 | 0.77 | 0.52 | 8.80 | 6 | 2 | 5 | eQTLs,C-HiC |
| rs3815521 | 122.9 | | 2.80E-07 | -0.13 | 0.56 | 0.62 | 0.40 | 0.77 | 0.52 | 2.62 | 6 | 13 | 28 | eQTLs,C-HiC |
| rs759403 | 121.8 | | 2.80E-07 | -0.13 | 0.56 | 0.63 | 0.40 | 0.78 | 0.52 | 2.90 | 6 | 14 | 23 | eQTLs,C-HiC |
| rs4997809 | 120.6 | | 2.80E-07 | -0.13 | 0.56 | 0.62 | 0.41 | 0.78 | 0.52 | 2.29 | 4 | 20 | 25 | eQTLs,C-HiC |
| rs4519489 | 118.0 | | 2.80E-07 | -0.13 | 0.56 | 0.62 | 0.40 | 0.77 | 0.52 | 1.90 | 3a | 18 | 36 | eQTLs,C-HiC |
| rs10803723 | 110.8 | | 2.80E-07 | -0.13 | 0.56 | 0.63 | 0.40 | 0.77 | 0.52 | 1.61 | 6 | 15 | 51 | eQTLs,C-HiC |
| rs4669599 | 109.6 | | 2.80E-07 | -0.13 | 0.56 | 0.62 | 0.40 | 0.77 | 0.52 | 1.83 | 4 | 13 | 41 | eQTLs,C-HiC |
| rs10181806 | 109.1 | | 2.80E-07 | -0.13 | 0.56 | 0.62 | 0.40 | 0.77 | 0.52 | 3.64 | 6 | 4 | 20 | eQTLs,C-HiC |
| rs728135 | 106.1 | | 2.80E-07 | -0.13 | 0.56 | 0.62 | 0.40 | 0.77 | 0.52 | 2.17 | 7 | 15 | 29 | eQTLs,C-HiC |
| rs6432122 | 105.2 | | 2.80E-07 | -0.13 | 0.56 | 0.62 | 0.40 | 0.77 | 0.52 | 6.58 | 4 | 5 | 4 | eQTLs,C-HiC |
| rs7587231 | 103.6 | | 2.80E-07 | -0.13 | . | . | . | . | . | 1.52 | 5 | 22 | 38 | eQTLs,C-HiC |
| rs4669593 | 102.1 | | 2.30E-07 | -0.13 | 0.56 | 0.62 | 0.40 | 0.78 | 0.51 | 1.86 | 4 | 13 | 38 | |
| rs9679122 | 97.9 | | 2.80E-07 | -0.13 | 0.56 | 0.62 | 0.40 | 0.77 | 0.52 | 2.97 | 4 | 4 | 24 | eQTLs,C-HiC |
| rs7566875 | 97.3 | | 2.80E-07 | -0.13 | 0.56 | 0.62 | 0.40 | 0.77 | 0.52 | 2.78 | 5 | 16 | 14 | eQTLs,C-HiC |
| rs6432107 | 92.3 | | 3.40E-07 | -0.13 | 0.57 | 0.63 | 0.40 | 0.80 | 0.52 | 1.85 | 5 | 11 | 34 | eQTLs,C-HiC |
| rs7587114 | 91.8 | | 2.80E-07 | -0.13 | . | . | . | . | . | 1.33 | 4 | 24 | 39 | eQTLs,C-HiC |
| rs759400 | 90.0 | | #N/A | #N/A | 0.75 | 0.75 | 0.52 | 0.81 | 0.79 | 1.08 | 1f | 14 | 68 | |
| rs10210859 | 87.2 | | 2.80E-07 | -0.13 | . | . | . | . | . | 1.26 | 5 | 14 | 51 | eQTLs,C-HiC |
| rs6721739 | 87.0 | | 2.80E-07 | -0.13 | 0.56 | 0.62 | 0.40 | 0.77 | 0.52 | 4.35 | 3a | 5 | 8 | eQTLs,C-HiC |
| rs6432126 | 79.0 | | 2.80E-07 | -0.13 | 0.56 | 0.62 | 0.40 | 0.78 | 0.52 | 1.20 | 5 | 20 | 40 | eQTLs,C-HiC |
| rs7566767 | 78.9 | | 2.80E-07 | -0.13 | . | . | . | . | . | 2.32 | 5 | 15 | 14 | eQTLs,C-HiC |
| rs1309 | 74.6 | | 2.80E-07 | -0.13 | 0.56 | 0.62 | 0.40 | 0.77 | 0.52 | 1.96 | 5 | 11 | 21 | eQTLs,C-HiC |
| rs11686969 | 73.3 | | 2.80E-07 | -0.13 | 0.56 | 0.62 | 0.40 | 0.77 | 0.52 | 1.44 | 7 | 6 | 38 | eQTLs,C-HiC |
| rs6432114 | 66.6 | | 2.80E-07 | -0.13 | 0.56 | 0.62 | 0.40 | 0.77 | 0.52 | 1.28 | 3a | 15 | 29 | eQTLs,C-HiC |
| rs6744854 | 65.4 | | 2.30E-06 | -0.12 | . | . | . | . | . | 2.42 | 7 | 6 | 16 | eQTLs,C-HiC |
| rs4470306 | 64.9 | | 2.80E-07 | -0.13 | 0.56 | 0.62 | 0.40 | 0.77 | 0.52 | 1.06 | 7 | 18 | 38 | eQTLs,C-HiC |
| rs10203785 | 64.7 | | 2.80E-07 | -0.13 | 0.56 | 0.63 | 0.40 | 0.77 | 0.52 | 4.98 | 6 | 3 | 5 | eQTLs,C-HiC |
| rs2357654 | 63.8 | | #N/A | #N/A | 0.75 | 0.75 | 0.53 | 0.81 | 0.79 | 1.52 | 5 | 9 | 26 | eQTLs,C-HiC |
| rs4669598 | 61.5 | | 2.80E-07 | -0.13 | 0.56 | 0.62 | 0.40 | 0.77 | 0.52 | 1.21 | 3a | 13 | 31 | eQTLs,C-HiC |
| rs6432111 | 60.2 | | 2.80E-07 | -0.13 | 0.56 | 0.62 | 0.40 | 0.77 | 0.52 | 1.40 | 6 | 12 | 26 | eQTLs,C-HiC |

| | | | | | | | | | | | | | | |
|---|---|---|---|---|---|---|---|---|---|---|---|---|---|---|
| rs6761678 | 60.0 | 2.80E-07 | -0.13 | 0.56 | 0.62 | 0.40 | 0.77 | 0.52 | 0.95 | 7 | 18 | 40 | eQTLs,C-HiC |
| rs10929682 | 59.4 | 2.80E-07 | -0.13 | 0.56 | 0.63 | 0.40 | 0.77 | 0.52 | 0.65 | 7 | 23 | 64 | eQTLs,C-HiC |
| rs7563816 | 58.7 | 2.80E-07 | -0.13 | 0.56 | 0.63 | 0.40 | 0.77 | 0.52 | 1.73 | 7 | 9 | 19 | eQTLs,C-HiC |
| rs4668691 | 58.2 | 2.80E-07 | -0.13 | 0.56 | 0.62 | 0.40 | 0.77 | 0.52 | 1.35 | 5 | 12 | 22 | eQTLs,C-HiC |
| rs728282 | 53.7 | 2.80E-07 | -0.13 | 0.56 | 0.62 | 0.40 | 0.77 | 0.52 | 3.58 | 5 | 4 | 6 | eQTLs,C-HiC |
| rs3814388 | 50.3 | 2.30E-07 | -0.13 | . | . | . | . | . | 1.20 | 2b | 9 | 25 | |
| rs4997810 | 49.7 | 2.80E-07 | -0.13 | 0.56 | 0.62 | 0.41 | 0.78 | 0.52 | 0.90 | 4 | 20 | 25 | eQTLs,C-HiC |
| rs6432125 | 49.0 | 2.80E-07 | -0.13 | 0.56 | 0.62 | 0.41 | 0.77 | 0.52 | 1.26 | 7 | 12 | 22 | eQTLs,C-HiC |
| rs6432120 | 47.4 | 2.80E-07 | -0.13 | . | . | . | . | . | 2.26 | 3a | 5 | 5 | eQTLs,C-HiC |
| rs6432119 | 47.2 | 2.80E-07 | -0.13 | 0.56 | 0.62 | 0.40 | 0.77 | 0.52 | 1.89 | 4 | 7 | 7 | eQTLs,C-HiC |
| rs4331474 | 46.4 | 1.60E-07 | -0.13 | 0.56 | 0.61 | 0.41 | 0.78 | 0.52 | 0.83 | 4 | 21 | 26 | eQTLs,C-HiC |
| rs13034061 | 44.7 | 2.80E-07 | -0.13 | 0.56 | 0.62 | 0.40 | 0.77 | 0.52 | 1.15 | 7 | 12 | 22 | eQTLs,C-HiC |
| rs4669596 | 43.0 | 2.80E-07 | -0.13 | 0.56 | 0.63 | 0.40 | 0.78 | 0.52 | 0.93 | 4 | 15 | 28 | eQTLs,C-HiC |
| rs7608498 | 41.3 | 2.80E-07 | -0.13 | 0.56 | 0.62 | 0.40 | 0.77 | 0.52 | 0.42 | 2b | 34 | 54 | eQTLs,C-HiC |
| rs10929687 | 38.8 | 2.80E-07 | -0.13 | 0.56 | 0.63 | 0.40 | 0.78 | 0.52 | 0.97 | 5 | 4 | 31 | eQTLs,C-HiC |
| rs12464817 | 34.7 | 2.80E-07 | -0.13 | 0.56 | 0.62 | 0.40 | 0.77 | 0.52 | 2.48 | 6 | 5 | 8 | |
| rs6432117 | 33.3 | 2.80E-07 | -0.13 | 0.56 | 0.62 | 0.40 | 0.77 | 0.52 | 1.07 | 5 | 6 | 20 | eQTLs,C-HiC |
| rs6721514 | 32.3 | 2.80E-07 | -0.13 | 0.56 | 0.62 | 0.40 | 0.77 | 0.52 | 1.90 | 4 | 4 | 7 | eQTLs,C-HiC |
| rs6432121 | 31.7 | 2.80E-07 | -0.13 | 0.56 | 0.62 | 0.40 | 0.77 | 0.52 | 1.76 | 4 | 5 | 5 | eQTLs,C-HiC |
| rs4371336 | 31.6 | 2.80E-07 | -0.13 | 0.56 | 0.62 | 0.40 | 0.77 | 0.52 | 0.59 | 7 | 20 | 25 | eQTLs,C-HiC |
| rs6759740 | 31.1 | 2.80E-07 | -0.13 | 0.56 | 0.62 | 0.40 | 0.77 | 0.52 | 1.24 | 6 | 6 | 14 | eQTLs,C-HiC |
| rs11683335 | 29.3 | 2.80E-07 | -0.13 | 0.56 | 0.63 | 0.40 | 0.78 | 0.52 | 0.68 | 5 | 6 | 32 | eQTLs,C-HiC |
| rs9679581 | 29.1 | 2.80E-07 | -0.13 | 0.56 | 0.62 | 0.40 | 0.77 | 0.52 | 0.88 | 4 | 3 | 25 | eQTLs,C-HiC |
| rs12692417 | 28.1 | 2.80E-07 | -0.13 | 0.56 | 0.62 | 0.40 | 0.77 | 0.52 | 0.94 | #N/A | 9 | 16 | eQTLs,C-HiC |
| rs6432109 | 27.9 | 2.80E-07 | -0.13 | 0.56 | 0.62 | 0.40 | 0.77 | 0.52 | 0.37 | 7 | 16 | 53 | eQTLs,C-HiC |
| rs6714413 | 27.9 | 1.60E-06 | -0.12 | 0.56 | 0.62 | 0.39 | 0.77 | 0.49 | 0.93 | 6 | 7 | 17 | eQTLs,C-HiC |
| rs6748036 | 26.1 | 2.80E-07 | -0.13 | 0.56 | 0.62 | 0.40 | 0.78 | 0.52 | 0.24 | 5 | 39 | 65 | eQTLs,C-HiC |
| rs7570118 | 24.9 | 2.80E-07 | -0.13 | 0.56 | 0.62 | 0.40 | 0.77 | 0.52 | 0.61 | 7 | 17 | 22 | eQTLs,C-HiC |
| rs6719701 | 23.1 | 2.80E-07 | -0.13 | 0.56 | 0.62 | 0.40 | 0.77 | 0.52 | 1.77 | 6 | 5 | 2 | eQTLs,C-HiC |
| rs2287062 | 23.0 | 2.80E-07 | -0.13 | 0.56 | 0.62 | 0.40 | 0.77 | 0.52 | 0.38 | 1f | 18 | 35 | eQTLs,C-HiC |
| rs12995159 | 20.6 | 2.80E-07 | -0.13 | 0.56 | 0.62 | 0.40 | 0.77 | 0.52 | 1.14 | 4 | 3 | 7 | eQTLs,C-HiC |
| rs7355649 | 20.5 | 5.20E-07 | -0.13 | 0.56 | 0.56 | 0.40 | 0.77 | 0.52 | 1.86 | 6 | 3 | 3 | eQTLs,C-HiC |
| rs2018512 | 20.2 | 1.60E-06 | -0.12 | 0.56 | 0.62 | 0.39 | 0.77 | 0.49 | 0.52 | #N/A | 11 | 23 | eQTLs,C-HiC |
| rs728283 | 19.9 | 2.80E-07 | -0.13 | 0.56 | 0.62 | 0.40 | 0.77 | 0.52 | 1.42 | 5 | 4 | 5 | eQTLs,C-HiC |
| rs4622695 | 18.3 | 2.80E-07 | -0.13 | 0.56 | 0.62 | 0.40 | 0.77 | 0.52 | 0.23 | 5 | 21 | 55 | eQTLs,C-HiC |
| rs6742448 | 17.9 | 1.60E-06 | -0.12 | 0.56 | 0.63 | 0.40 | 0.77 | 0.49 | 0.53 | 6 | 12 | 16 | eQTLs,C-HiC |
| rs6721510 | 17.5 | 2.80E-07 | -0.13 | 0.56 | 0.62 | 0.40 | 0.77 | 0.52 | 1.09 | 4 | 4 | 7 | eQTLs,C-HiC |
| rs6709466 | 17.4 | 3.20E-07 | -0.13 | 0.56 | 0.62 | 0.40 | 0.77 | 0.52 | 1.45 | #N/A | 5 | 2 | eQTLs,C-HiC |
| rs61447318 | 16.5 | #N/A | #N/A | . | . | . | . | . | 0.36 | #N/A | 8 | 27 | eQTLs,C-HiC |
| rs4284804 | 15.9 | 2.80E-07 | -0.13 | 0.56 | 0.63 | 0.40 | 0.77 | 0.52 | 0.30 | 5 | 11 | 39 | eQTLs,C-HiC |
| rs6706763 | 15.0 | 2.80E-07 | -0.13 | 0.56 | 0.62 | 0.40 | 0.77 | 0.52 | 0.75 | 7 | 6 | 9 | eQTLs,C-HiC |
| rs6744599 | 14.9 | 2.80E-07 | -0.13 | 0.56 | 0.62 | 0.40 | 0.77 | 0.52 | 0.39 | 6 | 13 | 19 | eQTLs,C-HiC |
| rs2110778 | 13.6 | 2.80E-07 | -0.13 | 0.56 | 0.62 | 0.40 | 0.77 | 0.52 | 0.91 | #N/A | 5 | 1 | eQTLs,C-HiC |
| rs12472807 | 13.0 | 3.90E-06 | -0.12 | 0.56 | 0.63 | 0.39 | 0.77 | 0.49 | 0.50 | 5 | 7 | 10 | eQTLs,C-HiC |
| rs6712304 | 12.5 | 2.80E-07 | -0.13 | 0.56 | 0.62 | 0.40 | 0.77 | 0.52 | 0.15 | 5 | 16 | 28 | eQTLs,C-HiC |
| rs11683148 | 12.0 | 2.80E-07 | -0.13 | 0.56 | 0.62 | 0.40 | 0.77 | 0.52 | 0.18 | 7 | 6 | 38 | eQTLs,C-HiC |
| rs61424402 | 8.9 | #N/A | #N/A | 0.56 | 0.63 | 0.39 | 0.74 | 0.52 | 0.23 | #N/A | 11 | 23 | eQTLs,C-HiC |
| rs12995602 | 8.9 | 2.80E-07 | -0.13 | 0.56 | 0.62 | 0.40 | 0.77 | 0.52 | 0.33 | 4 | 7 | 9 | eQTLs,C-HiC |
| rs6750891 | 8.9 | 2.80E-07 | -0.13 | . | . | . | . | . | 0.12 | 4 | 27 | 44 | eQTLs,C-HiC |
| rs6432118 | 8.2 | 2.80E-07 | -0.13 | 0.56 | 0.62 | 0.40 | 0.77 | 0.52 | 0.30 | 7 | 3 | 19 | eQTLs,C-HiC |
| rs9287720 | 7.5 | 2.80E-07 | -0.13 | 0.56 | 0.62 | 0.40 | 0.77 | 0.52 | 0.34 | 7 | 3 | 14 | eQTLs,C-HiC |
| rs6432115 | 6.8 | 2.80E-07 | -0.13 | 0.56 | 0.62 | 0.40 | 0.77 | 0.52 | 0.57 | 5 | 3 | 2 | eQTLs,C-HiC |
| rs10203664 | 6.0 | 2.80E-07 | -0.13 | 0.56 | 0.62 | 0.40 | 0.77 | 0.52 | 0.43 | 7 | 2 | 7 | eQTLs,C-HiC |
| rs6751075 | 5.9 | 2.80E-07 | -0.13 | 0.56 | 0.63 | 0.40 | 0.77 | 0.52 | 0.11 | 5 | 14 | 37 | eQTLs,C-HiC |
| rs10167676 | 5.9 | #N/A | #N/A | 0.56 | 0.62 | 0.40 | 0.77 | 0.52 | 0.35 | #N/A | 2 | 9 | eQTLs,C-HiC |
| rs6432116 | 5.4 | 2.80E-07 | -0.13 | 0.56 | 0.62 | 0.40 | 0.77 | 0.52 | 0.23 | 4 | 8 | 8 | eQTLs,C-HiC |
| rs2110776 | 4.5 | 2.80E-07 | -0.13 | 0.56 | 0.62 | 0.40 | 0.77 | 0.52 | 0.30 | 5 | 6 | 1 | eQTLs,C-HiC |
| rs13003763 | 3.4 | 2.30E-07 | -0.13 | 0.56 | 0.61 | 0.40 | 0.78 | 0.51 | 0.26 | 5 | 2 | 11 | |
| rs715246 | 0.5 | 2.80E-07 | -0.13 | 0.56 | 0.62 | 0.40 | 0.77 | 0.52 | 0.01 | 5 | 7 | 27 | eQTLs,C-HiC |
| rs2287061 | 0.2 | 2.80E-07 | -0.13 | 0.56 | 0.62 | 0.40 | 0.77 | 0.52 | 0.00 | 5 | 18 | 34 | eQTLs,C-HiC |

# Table S8 Scoring of eQTL SNPs for rs77575195 LD block

| RSID | Score | GWAS | Nerve - Tibial p-value | Nerve - Tibial NES | AMR | EAS | SAS | AFR | EUR | CADD | RegulomeDB | ENCODE Hits | ROADMAP Hits | GeneHancer |
|---|---|---|---|---|---|---|---|---|---|---|---|---|---|---|
| rs77575195 | 104.368 | | 8.30E-09 | 0.16 | 0.1873 | 0.128 | 0.1207 | 0.0113 | 0.2773 | 2.372 | 4 | 7 | 35 | #N/A |
| rs77767350 | 797.552 | | 1.50E-08 | 0.16 | 0.1859 | 0.127 | 0.1227 | 0.0113 | 0.2744 | 7.121 | 4 | 29 | 78 | eQTLs,C-HiC |
| rs76761022 | 369.936 | | 1.20E-08 | 0.16 | 0.1859 | 0.128 | 0.1227 | 0.0333 | 0.2763 | 3.303 | 3a | 54 | 53 | eQTLs,C-HiC |
| rs113441467 | 332.674 | | 1.00E-08 | 0.16 | 0.1859 | 0.128 | 0.1227 | 0.0121 | 0.2744 | 4.057 | 3a | 18 | 57 | eQTLs,C-HiC |
| rs1012580 | 265.85 | | 3.60E-08 | 0.16 | 0.1873 | 0.124 | 0.1227 | 0.0113 | 0.2833 | 4.09 | 4 | 29 | 31 | eQTLs,C-HiC |
| rs11903800 | 247.028 | | 1.70E-06 | 0.13 | 0.2205 | 0.1429 | 0.136 | 0.3472 | 0.3002 | 2.988 | 5 | 26 | 50 | eQTLs,C-HiC |
| rs75295272 | 232.416 | | 1.00E-08 | 0.16 | 0.1859 | 0.128 | 0.1227 | 0.0113 | 0.2744 | 6.456 | 6 | 11 | 19 | eQTLs,C-HiC |
| rs74979008 | 228.285 | | 1.00E-08 | 0.16 | 0.1844 | 0.128 | 0.1227 | 0.0113 | 0.2744 | 3.265 | 5 | 12 | 53 | eQTLs,C-HiC |
| rs17455388 | 218.018 | | 9.60E-08 | 0.16 | 0.183 | 0.124 | 0.1237 | 0.0098 | 0.2694 | 3.254 | 5 | 32 | 29 | eQTLs,C-HiC |
| rs111861993 | 166.14 | | 4.50E-08 | 0.15 | 0.1873 | 0.124 | 0.1237 | 0.0113 | 0.2833 | 2.556 | 5 | 17 | 43 | eQTLs,C-HiC |
| rs35516187 | 161.211 | | 1.50E-08 | 0.16 | 0.183 | 0.128 | 0.1227 | 0.0113 | 0.2744 | 1.479 | 7 | 32 | 73 | eQTLs,C-HiC |
| rs7603803 | 117.208 | | 3.60E-08 | 0.16 | . | . | . | . | . | 2.392 | 5 | 9 | 35 | eQTLs,C-HiC |
| rs114684284 | 85.6 | | #N/A | #N/A | 0.1873 | 0.124 | 0.1237 | 0.0212 | 0.2833 | 2.675 | 6 | 3 | 24 | eQTLs,C-HiC |
| rs78463923 | 66.56 | | 1.00E-08 | 0.16 | 0.1844 | 0.128 | 0.1227 | 0.0113 | 0.2753 | 1.04 | 6 | 23 | 41 | #N/A |
| rs7603290 | 64.752 | | #N/A | #N/A | 0.8112 | 0.876 | 0.8763 | 0.9493 | 0.7167 | 1.349 | #N/A | 10 | 31 | eQTLs,C-HiC |
| rs74799085 | 53.872 | | 1.20E-08 | 0.16 | 0.1859 | 0.128 | 0.1227 | 0.0333 | 0.2773 | 0.518 | 5 | 50 | 52 | eQTLs,C-HiC |
| rs149307648 | 45.856 | | 7.10E-08 | 0.15 | 0.1873 | 0.123 | 0.1247 | 0.0113 | 0.2833 | 5.732 | 5 | 1 | 6 | #N/A |
| rs77047837 | 35.786 | | 1.70E-08 | 0.16 | . | . | . | . | . | 0.617 | 5 | 9 | 45 | eQTLs,C-HiC |
| rs77909024 | 35.232 | | 1.00E-08 | 0.16 | 0.1859 | 0.127 | 0.1227 | 0.0113 | 0.2744 | 2.202 | 7 | 3 | 8 | eQTLs,C-HiC |
| rs78979433 | 28.458 | | 1.00E-08 | 0.16 | 0.1844 | 0.128 | 0.1227 | 0.0113 | 0.2744 | 0.459 | #N/A | 23 | 32 | eQTLs,C-HiC |
| rs764526 | 26.892 | | 4.50E-08 | 0.15 | 0.1873 | 0.124 | 0.1237 | 0.0113 | 0.2833 | 0.249 | 4 | 40 | 62 | eQTLs,C-HiC |
| rs115591000 | 22.219 | | 5.90E-08 | 0.15 | 0.1873 | 0.124 | 0.1237 | 0.0113 | 0.2833 | 1.307 | 7 | 1 | 11 | eQTLs,C-HiC |
| rs77793365 | 10.735 | | 5.70E-08 | 0.15 | 0.1873 | 0.124 | 0.1237 | 0.0129 | 0.2833 | 2.147 | 5 | 3 | 2 | #N/A |
| rs1546026 | 7.774 | | 3.60E-08 | 0.16 | 0.1873 | 0.124 | 0.1237 | 0.0129 | 0.2833 | 3.887 | 5 | 1 | 1 | #N/A |
| rs75419580 | 7.192 | | 1.20E-07 | 0.15 | 0.1873 | 0.126 | 0.1227 | 0.0121 | 0.2833 | 0.058 | 3a | 41 | 76 | eQTLs,C-HiC |
| rs76923771 | 5.324 | | 1.00E-08 | 0.16 | 0.1844 | 0.128 | 0.1227 | 0.0113 | 0.2744 | 0.004 | 4 | 24 | 52 | eQTLs,C-HiC |
| rs78712844 | 2.496 | | 1.00E-08 | 0.16 | 0.1844 | 0.128 | 0.1227 | 0.0113 | 0.2753 | 0.064 | 5 | 15 | 21 | eQTLs,C-HiC |
| rs113291671 | 2.484 | | 4.50E-08 | 0.15 | . | . | . | . | . | 0.036 | 5 | 17 | 45 | eQTLs,C-HiC |
| rs2287059 | 0 | | 1.00E-08 | 0.16 | 0.1844 | 0.128 | 0.1227 | 0.0113 | 0.2744 | 0 | 7 | 1 | 1 | eQTLs,C-HiC |

**Table S9 IEU open GWAS project PheWAS returns for top SNPs of Geno2MP.** Traits for rs138359527 (ODC G84R) are colored red.

| SNP | ID | Sub category | Trait | Position | P | Beta | SE | N | CHR | EA | NEA | EAF |
|---|---|---|---|---|---|---|---|---|---|---|---|---|
| rs138359527 | ubm-a-2869 | Psychiatric / neurological | a2009s rh G Ins lg&S cent ins area | 10584626 | 0.000562 | 0.451 | 0.1307 | 7916 | 2 | T | C | 0.002551 |
| rs138359527 | ukb-a-109 | NA | Non-cancer illness code self-reported: hiatus hernia | 10584626 | 0.000626 | 0.012867 | 0.003762 | 337159 | 2 | T | C | 0.00239 |
| rs138359527 | ukb-a-544 | NA | Diagnoses - main ICD10: K20 Oesophagitis | 10584626 | 0.000675 | 0.008427 | 0.002479 | 337199 | 2 | T | C | 0.00239 |
| rs138359527 | ubm-a-2583 | Psychiatric / neurological | NET100 1427 | 10584626 | 0.000813 | 0.5165 | 0.1542 | 7916 | 2 | T | C | 0.002551 |
| rs138359527 | ubm-a-1136 | Psychiatric / neurological | NET25 0190 | 10584626 | 0.000955 | -0.5066 | 0.1532 | 7916 | 2 | T | C | 0.002551 |
| rs61754475 | eqtl-a-ENSG00000172059 | NA | ENSG00000172059 | 10268999 | 1.34E-12 | -0.33828 | 0.047714 | 7967 | 2 | T | C | 0.015753 |
| rs61754475 | eqtl-a-ENSG00000115756 | NA | ENSG00000115756 | 10268999 | 1.11E-06 | 0.232671 | 0.047759 | 7967 | 2 | T | C | 0.015753 |
| rs61754475 | eqtl-a-ENSG00000115750 | NA | ENSG00000115750 | 10268999 | 0.000123 | 0.183471 | 0.047774 | 7218 | 2 | T | C | 0.015753 |
| rs61754475 | ubm-a-1286 | Psychiatric / neurological | NET100 0130 | 10268999 | 0.000186 | -0.337 | 0.0901 | 7916 | 2 | T | C | 0.00819 |
| rs61754475 | ubm-a-2101 | Psychiatric / neurological | NET100 0945 | 10268999 | 0.000324 | 0.3252 | 0.0904 | 7916 | 2 | T | C | 0.00819 |
| rs61754475 | prot-a-1438 | Immune system | Interferon omega-1 | 10268999 | 0.000372 | -0.4582 | 0.1287 | 3301 | 2 | T | C | 0.01108 |
| rs61754475 | prot-a-2836 | Immune system | Serine palmitoyltransferase 1 | 10268999 | 0.000589 | -0.4425 | 0.1288 | 3301 | 2 | T | C | 0.01108 |
| rs61754475 | ubm-a-594 | Psychiatric / neurological | IDP dMRI TBSS ISOVF Cerebral peduncle R | 10268999 | 0.000617 | -0.2696 | 0.0788 | 7916 | 2 | T | C | 0.00819 |
| rs61754475 | prot-a-170 | Immune system | Arrestin domain-containing protein 3 | 10268999 | 0.000646 | 0.4392 | 0.1288 | 3301 | 2 | T | C | 0.01108 |
| rs61754475 | ukb-a-237 | NA | Pack years of smoking PREVIEW ONLY | 10268999 | 0.000805 | -0.08429 | 0.025151 | 101726 | 2 | T | C | 0.007998 |
| rs61754475 | prot-a-2407 | Immune system | Pregnancy-specific beta-1-glycoprotein 8 | 10268999 | 0.000832 | -0.43 | 0.1288 | 3301 | 2 | T | C | 0.01108 |
| rs61754475 | ukb-a-238 | NA | Pack years adult smoking as proportion of life span exposed to smoking PREVIEW ONLY | 10268999 | 0.000839 | -0.08428 | 0.025237 | 101726 | 2 | T | C | 0.007998 |
| rs138712410 | ukb-a-125 | NA | Treatment/medication code: kapake tablet | 10929938 | 5.16E-06 | 0.001417 | 0.000311 | 337159 | 2 | A | G | 0.004518 |
| rs138712410 | ubm-a-2724 | Psychiatric / neurological | DKTatlas lh lateralorbitofrontal area | 10929938 | 3.31E-05 | -0.3377 | 0.0813 | 7916 | 2 | A | G | 0.004401 |
| rs138712410 | ubm-a-144 | Psychiatric / neurological | IDP T1 FAST ROIs L cerebellum crus I | 10929938 | 8.51E-05 | -0.408 | 0.1037 | 7916 | 2 | A | G | 0.004401 |
| rs138712410 | ukb-d-1468_5 | NA | Cereal type: Other (e.g. Cornflakes, Frosties) | 10929938 | 0.000209 | -0.02882 | 0.007774 | 299898 | 2 | A | G | 0.004586 |
| rs138712410 | ukb-d-1428_1 | NA | Spread type: Butter/spreadable butter | 10929938 | 0.000623 | -0.02961 | 0.008654 | 360291 | 2 | A | G | 0.004595 |
| rs138712410 | ubm-a-2466 | Psychiatric / neurological | NET100 1310 | 10929938 | 0.000851 | 0.4014 | 0.1202 | 7916 | 2 | A | G | 0.004401 |
| rs138712410 | ukb-a-165 | NA | Treatment/medication code: nicorandil | 10929938 | 0.000851 | 0.003519 | 0.001055 | 337159 | 2 | A | G | 0.004518 |
| rs190385156 | prot-a-2805 | Immune system | Protein SPACA7 | 11273459 | 0.000155 | 0.3614 | 0.0956 | 3301 | 2 | T | C | 0.01831 |
| rs190385156 | ubm-a-3043 | Psychiatric / neurological | DKTatlas rh lingual thickness | 11273459 | 0.000347 | -0.2059 | 0.0576 | 7916 | 2 | T | C | 0.019227 |
| rs190385156 | ubm-a-605 | Psychiatric / neurological | IDP dMRI TBSS ISOVF Superior corona radiata L | 11273459 | 0.000501 | -0.1753 | 0.0504 | 7916 | 2 | T | C | 0.019227 |
| rs190385156 | ukb-a-446 | NA | Blood clot DVT bronchitis emphysema asthma rhinitis eczema allergy diagnosed by doctor: Asthma | 11273459 | 0.000589 | 0.01055 | 0.00307 | 336782 | 2 | T | C | 0.017863 |
| rs190385156 | ubm-a-3086 | Psychiatric / neurological | a2009s rh G oc-temp med-Lingual thickness | 11273459 | 0.000661 | -0.1959 | 0.0575 | 7916 | 2 | T | C | 0.019227 |
| rs190385156 | ubm-a-1854 | Psychiatric / neurological | NET100 0698 | 11273459 | 0.000676 | 0.2042 | 0.06 | 7916 | 2 | T | C | 0.019227 |
| rs190385156 | prot-a-1778 | Immune system | Low-density lipoprotein receptor-related protein 1B | 11273459 | 0.000813 | 0.3202 | 0.0956 | 3301 | 2 | T | C | 0.01831 |
| rs190385156 | finn-a-L12_INFECT_SKIN_NAS | NA | Other and unspecified local infections of skin and subcutaneous tissue | 11133333 | 0.000906 | -1.264 | 0.3809 | | 2 | C | T | 0.98903 |
| rs143556651 | ubm-a-3005 | Psychiatric / neurological | a2009s lh S circular insula ant thickness | 10904524 | 7.76E-05 | 0.6 | 0.1518 | 7916 | 2 | T | C | 0.002499 |
| rs143556651 | ukb-b-13231 | NA | Place of birth in UK - east co-ordinate | 10904524 | 0.0001 | 0.075512 | 0.019412 | 434576 | 2 | T | C | 0.002399 |
| rs143556651 | ukb-a-157 | NA | Treatment/medication code: liothyronine | 10904524 | 0.000111 | 0.001581 | 0.000409 | 337159 | 2 | T | C | 0.002347 |
| rs143556651 | ukb-a-469 | NA | Pain type(s) experienced in last month: Headache | 10904524 | 0.000205 | 0.037803 | 0.010181 | 336650 | 2 | T | C | 0.002347 |
| rs143556651 | ubm-a-2942 | Psychiatric / neurological | DKTatlas lh parsopercularis thickness | 10904524 | 0.000219 | 0.5334 | 0.1443 | 7916 | 2 | T | C | 0.002499 |
| rs143556651 | ubm-a-2970 | Psychiatric / neurological | a2009s lh G front inf-Opercular thickness | 10904524 | 0.000234 | 0.5387 | 0.1465 | 7916 | 2 | T | C | 0.002499 |
| rs143556651 | ubm-a-2308 | Psychiatric / neurological | NET100 1152 | 10904524 | 0.000245 | -0.586 | 0.1599 | 7916 | 2 | T | C | 0.002499 |
| rs143556651 | ubm-a-1257 | Psychiatric / neurological | NET100 0101 | 10904524 | 0.000363 | -0.5822 | 0.1632 | 7916 | 2 | T | C | 0.002499 |
| rs143556651 | ukb-a-391 | NA | Average weekly intake of other alcoholic drinks | 10904524 | 0.000543 | 0.033674 | 0.009736 | 79291 | 2 | T | C | 0.002347 |
| rs143556651 | ubm-a-3129 | Psychiatric / neurological | a2009s rh S parieto occipital thickness | 10904524 | 0.000724 | 0.4971 | 0.1469 | 7916 | 2 | T | C | 0.002499 |
| rs143556651 | ubm-a-2001 | Psychiatric / neurological | NET100 0845 | 10904524 | 0.000891 | -0.5454 | 0.164 | 7916 | 2 | T | C | 0.002499 |
| rs143556651 | ubm-a-3055 | Psychiatric / neurological | DKTatlas rh precuneus thickness | 10904524 | 0.000891 | 0.4838 | 0.1457 | 7916 | 2 | T | C | 0.002499 |

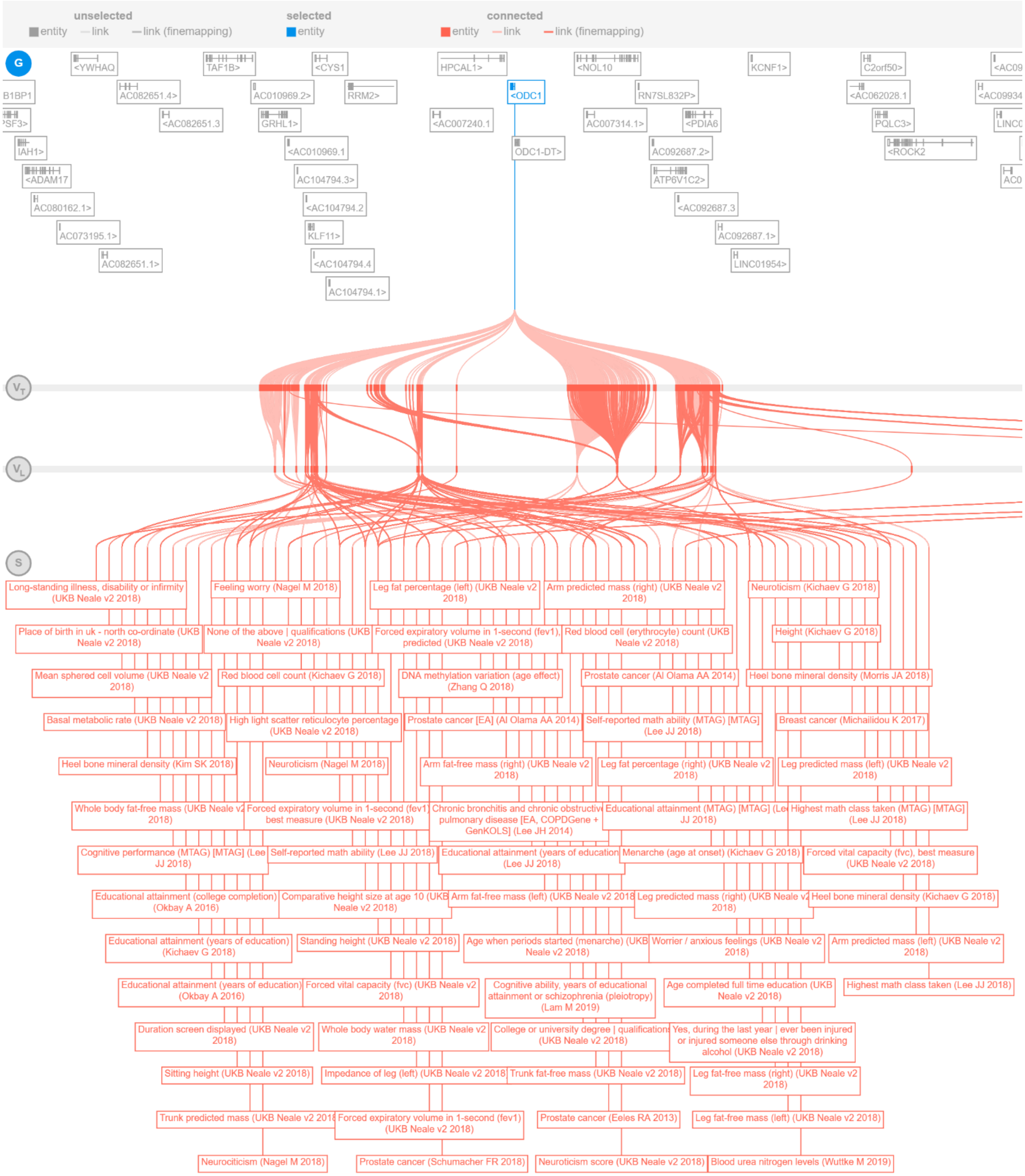


**Figure S1 Phenotypes associated with *ODC1* genomic region from Open Targets Genetics.**

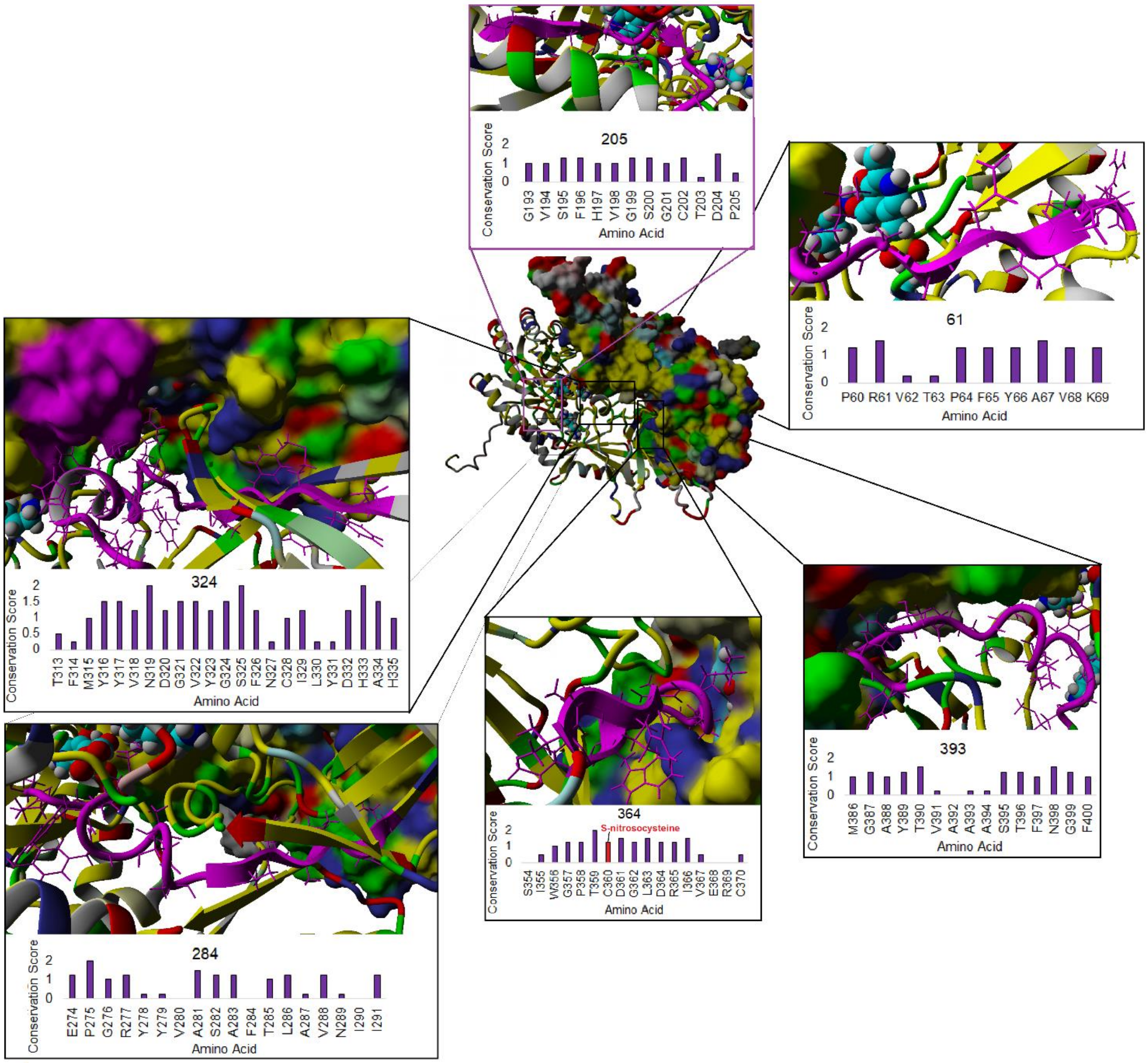


**Figure S2 Sequence and structural evolution of ODC.** Structure of ODC as a dimer (one unit shown with surface plot and other as secondary structure with amino acids colored based on ConSurf mapping (yellow- conserved hydrophobic, green- conserved hydrophilic, red- conserved acidic, blue- conserved basic, gray- not conserved). The six top motifs are shown with zoomed in view, colored magenta, and conservation shown below.

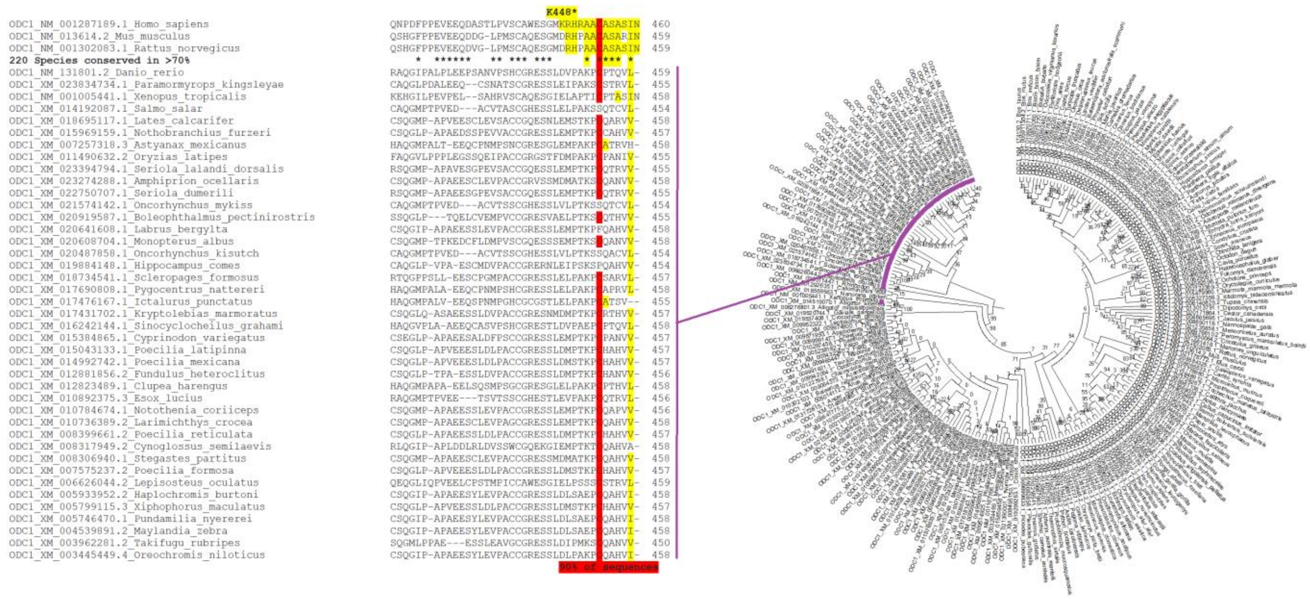

**Figure S3 ODC lipidation site.** The predicted farnesylation site falls at Cys 354, which is conserved (red) in all but a handful of fish and invertebrate genomes (magenta). The phylogenic tree shows the bootstrap consensus of 1000 runs, listing the clustering of trees at each node. The high conservation of flanking amino acids (yellow) contribute to the 12.669 score by GPS-Lipid 1.0 for non-consensus S-Farnesylation and a 3.64 score for non-consensus S-Geranylgeranylation.

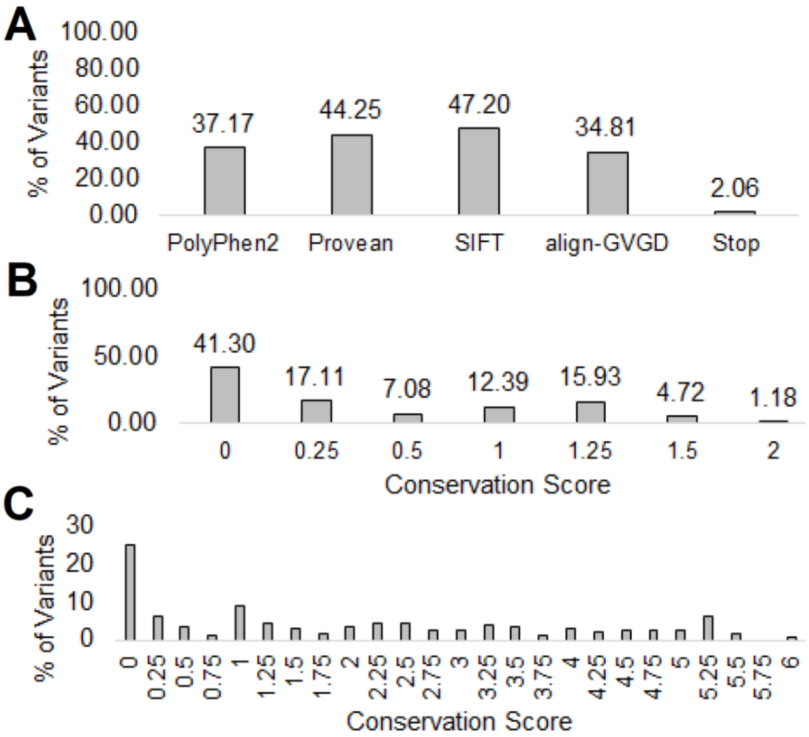


**Figure S4 Human variation in *ODC1*. A-C)** A total of 338 variants seen for *ODC1* in gnomAD, TOPMed (BRAVO), COSMIC, and ClinVar shown for those predicted damaging in several publicly available tools (**A**), our conservation scores (**B**), or the combined metrics of all tools (**C**).

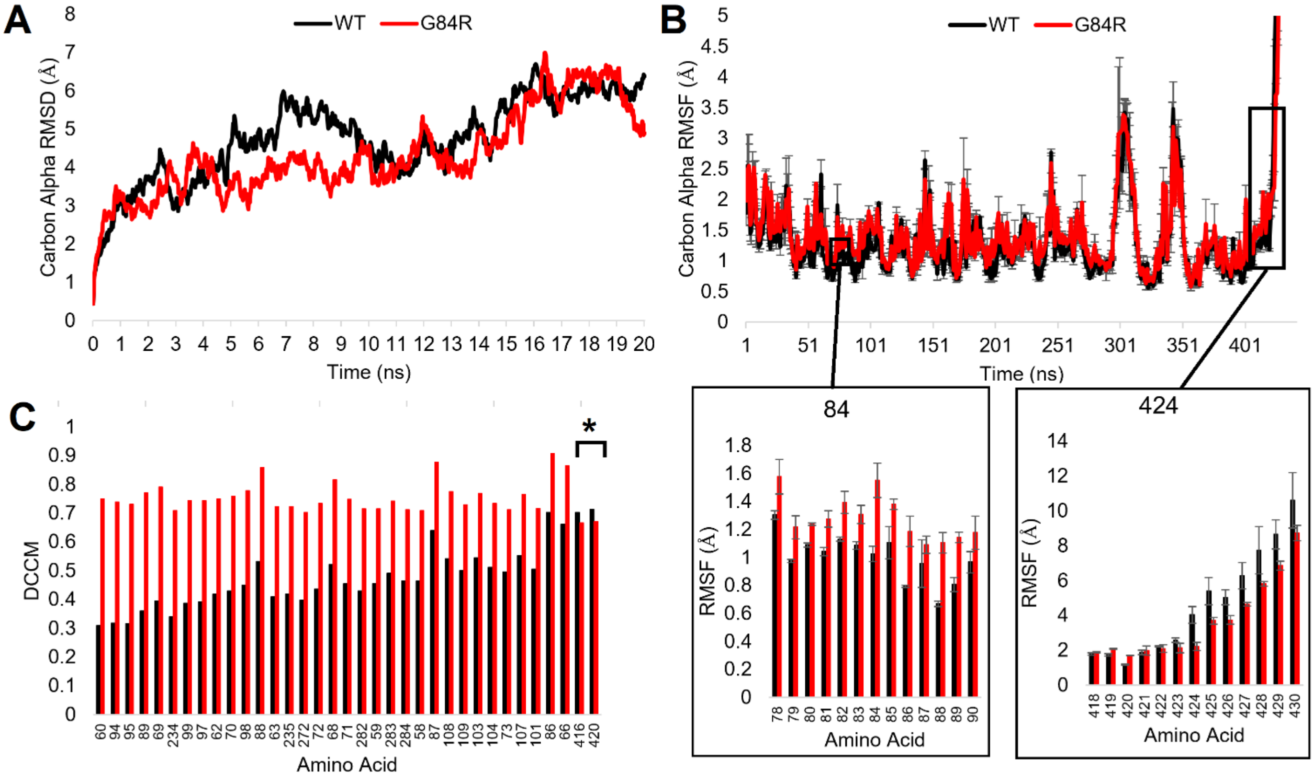


**Figure S5 Molecular dynamic simulations (mds) for ODC and ODC G84R**. **A)** Overall protein movement (root mean square deviation, RMSD) over 20 nanoseconds showing that both wild type (WT, black) and G84R (red) proteins reach relative equilibrium over the simulation. **B)** Amino acid movement shown as root mean squared fluctuation (RMSF) for WT (black) or G84R (red). Shown below is a zoom in of amino acid 84 and 424 regions. Error bars represent the standard error of the mean of the two ODC units of the dimer structure. **C)** Amino acid correlations throughout the 20 nanoseconds of simulation (dynamics cross-correlation matrix information, DCCM) for amino acids with WT amino acid 84 (black) or G84R (red). Shown are those sites with at least one of the two >0.7 correlation and are in order of largest differences. Only two amino acids show increased stability in wild type (*), while G84R is higher for 33 amino acids by 0.2 or more.

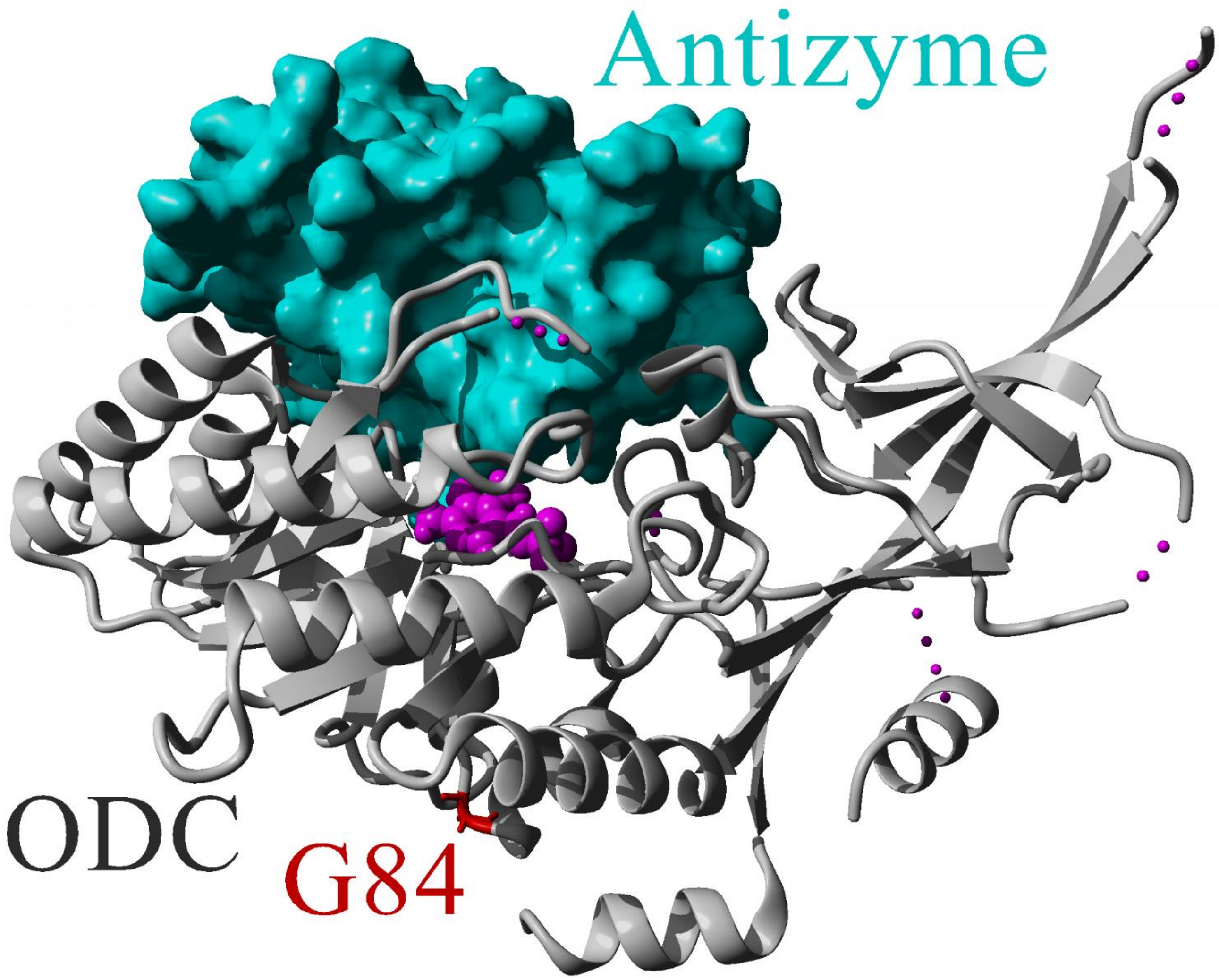


**Figure S6 ODC G84 location relative to antizyme binding**. The PDB file 4ZGY of ODC (gray) interacting with antizyme (cyan) with the G84 marked in red.

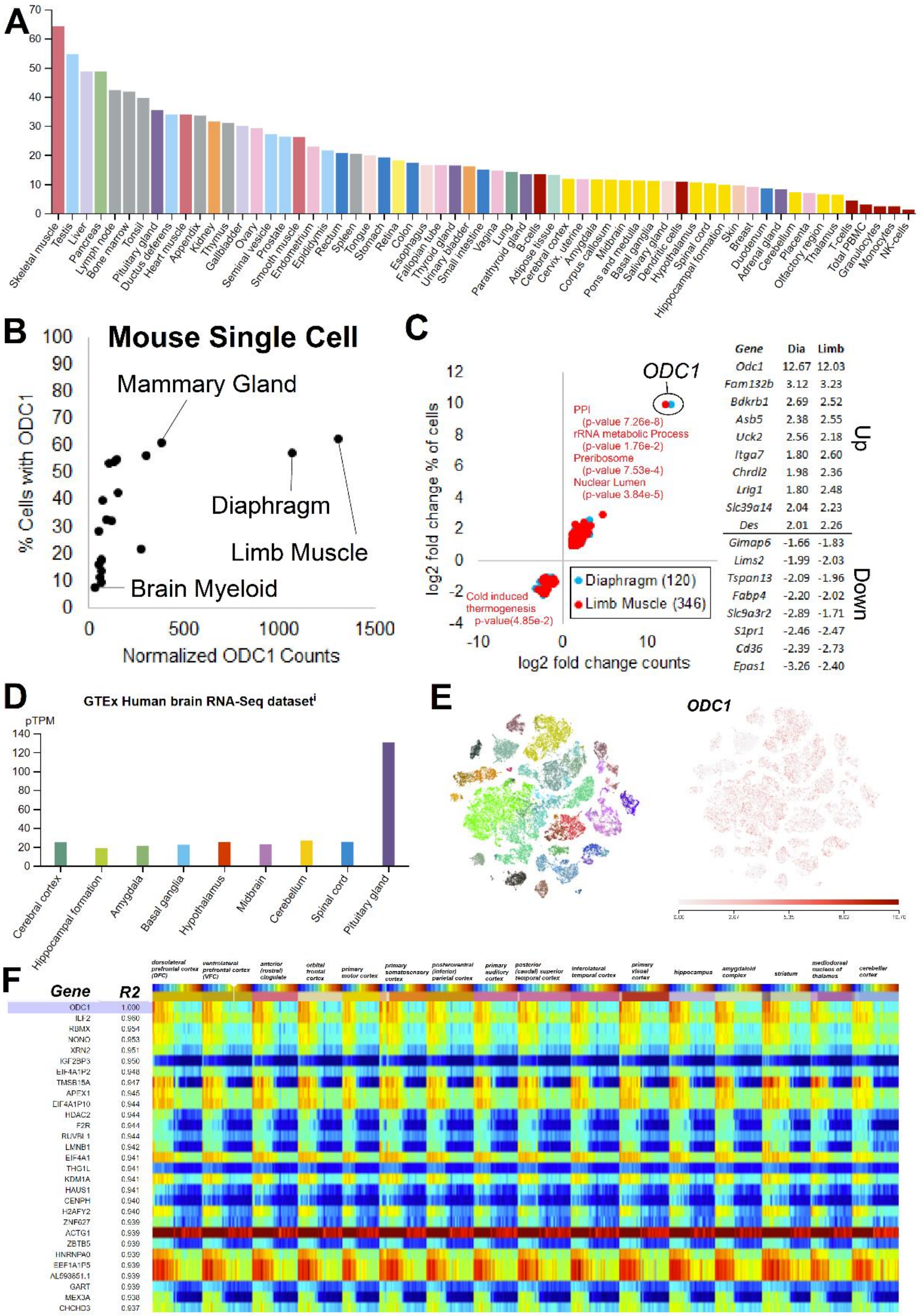


**Figure S7 Expression analysis of *ODC1*. A**) Expression of *ODC1* from Human Protein Atlas. **B)** Dissection of mouse single cell datasets for *ODC1* showing the average normalized counts per cell (x-axis) and the percent of cells *ODC1* is expressed in (y-axis). **C)** Clustering of genes following single cell levels of *ODC1* in diaphragm (blue) and limb muscle (red), identifying overlapping genes (right) and GO enrichment for positive or negative *ODC1* expressing cells (red text). **D)** Expression of *ODC1* in human brain regions from GTEx. **E)** *ODC1* single cell expression from Allen Brain Atlas Human multiple cortical areas- SMART-seq. On the left is coloring based on cell grouping of the 49,495 nuclei and on the right is expression of *ODC1* (red) based on the scale below. **F)** *ODC1* expression from human samples of Allen Brain Atlas Developmental Transcriptome over various ages and brain regions with highly correlated genes to *ODC1* over all brain regions shown below.

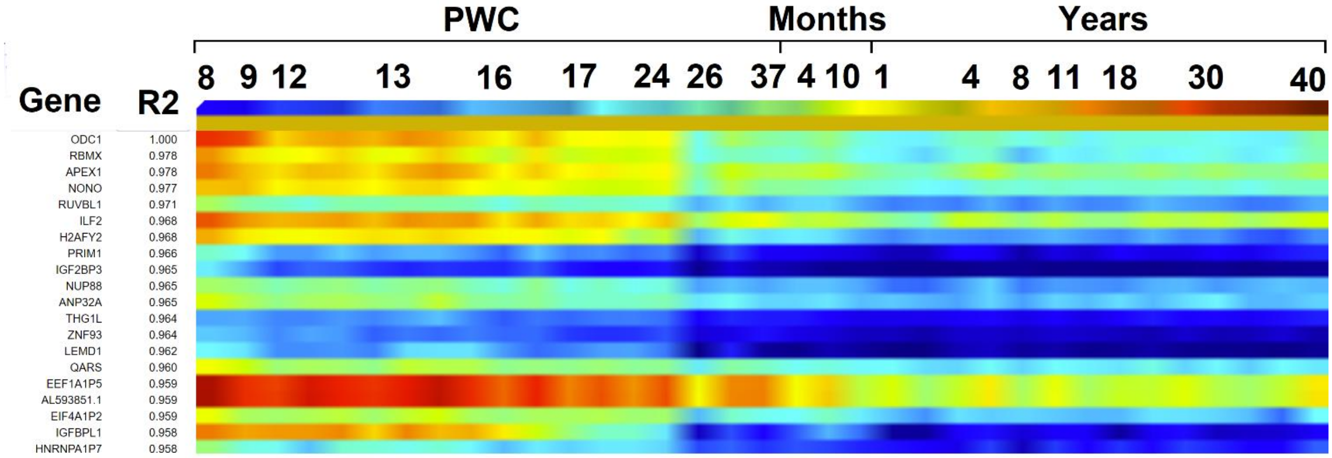


**Figure S8 Brain developmental time analysis of *ODC1* for dorsolateral prefrontal cortex (DFC).** *ODC1* expression from human samples of Allen Brain Atlas Developmental Transcriptome over various ages (left to right increase) for dorsolateral prefrontal cortex, with highly correlated genes to *ODC1* within this tissue shown below.

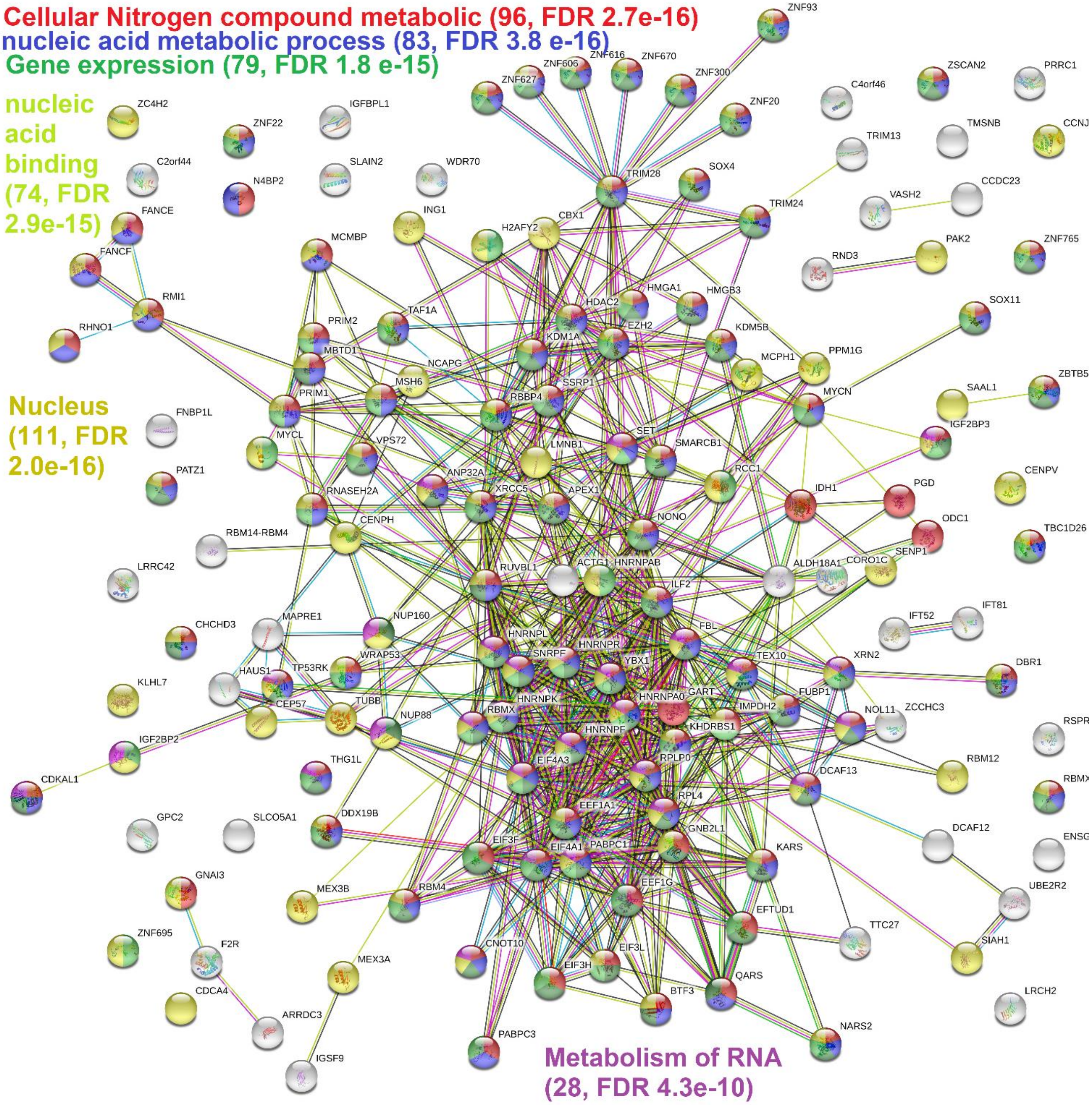


**Figure S9 *ODC1* brain positive correlated gene network.** 213 genes with greater than 0.9 $R^2$ correlation in expression over all brain regions of the Allen Brain Atlas Developmental Transcriptome shown as a STRING protein network with significantly elevated gene ontology terms labeled in various colors.

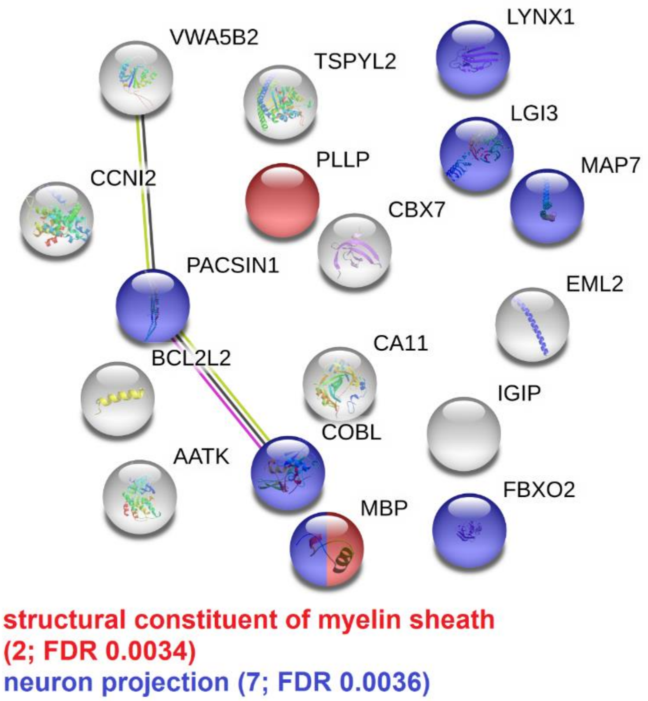


**Figure S10 *ODC1* brain negative correlated gene network.** 17 genes with less than -0.9 $R^2$ correlation in expression over all brain regions of the Allen Brain Atlas Developmental Transcriptome shown as a STRING protein network with significantly elevated gene ontology terms labeled in various colors.

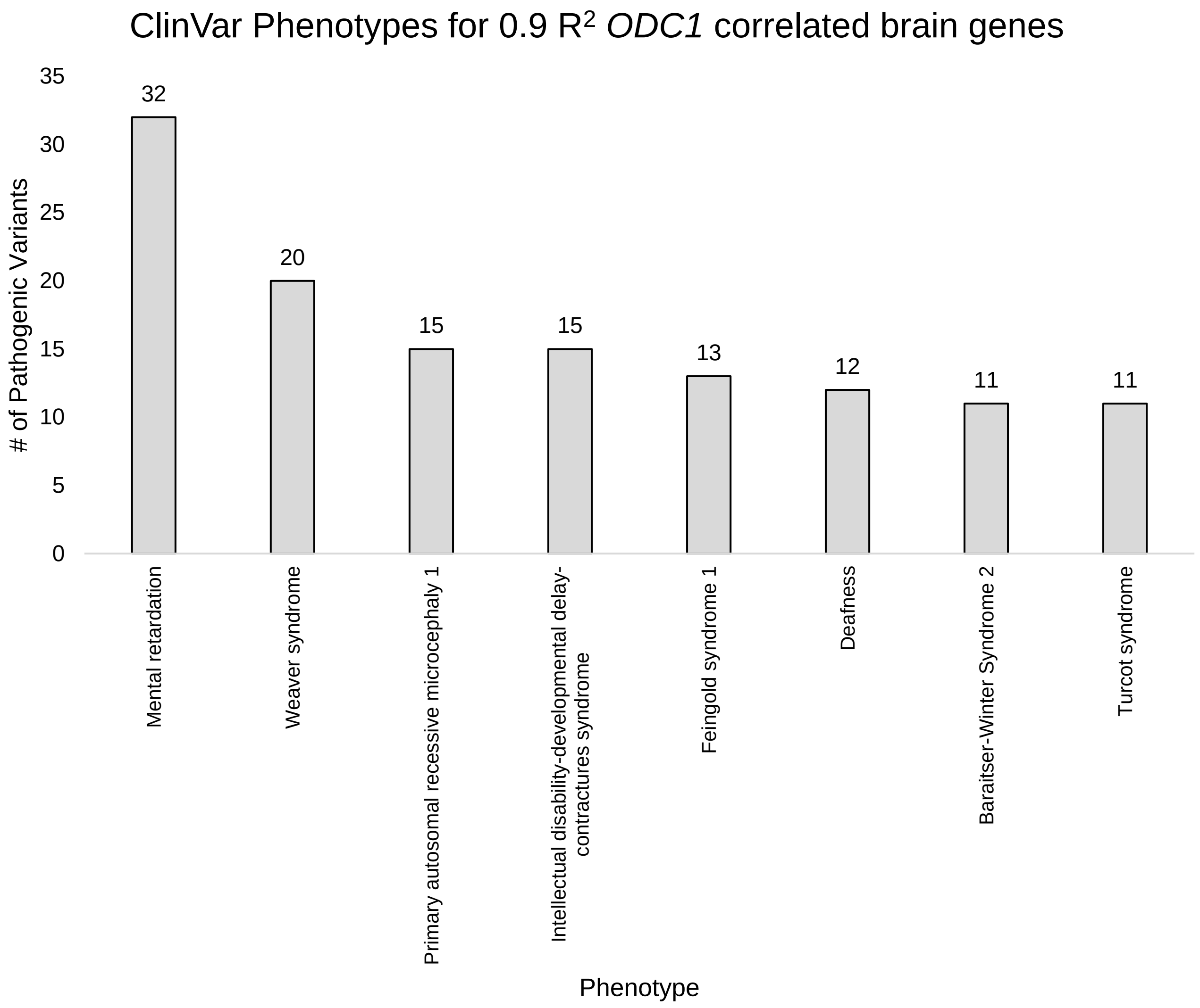


**Figure S11 ClinVar of 0.9 $R^2$ *ODC1* correlated genes from Allen Brain Atlas Developmental Transcriptome**. Genes from Figure S8 were extracted from ClinVar pathogenic annotations, pulling clinical annotations. Eight different neurological phenotypes are reported with the number of variants linked to each term shown.

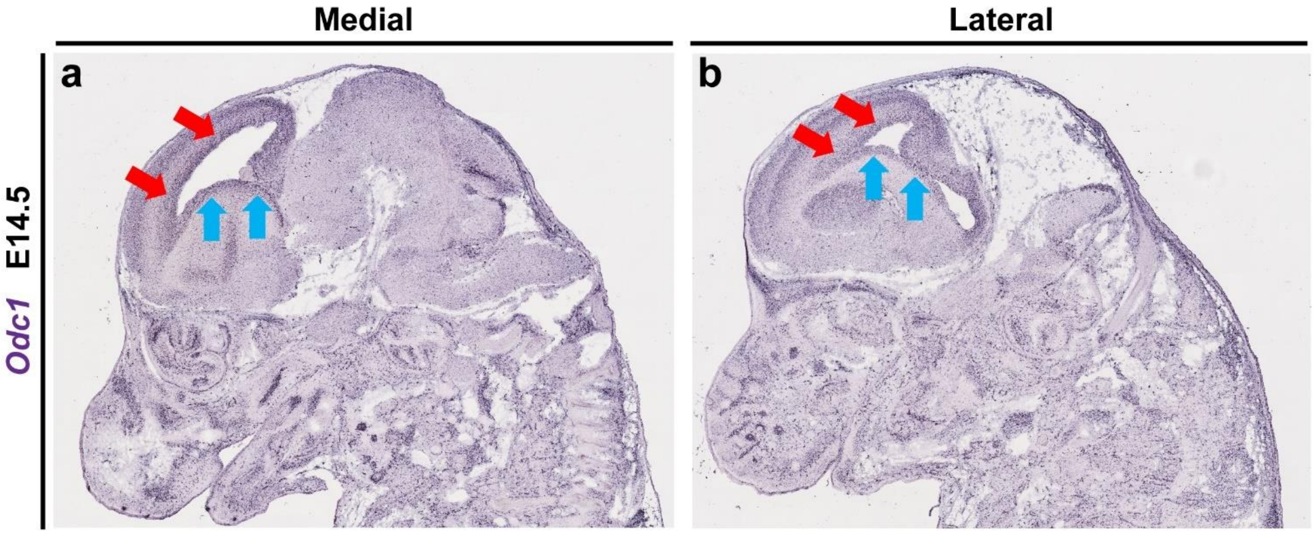


**Figure S12 *Odc1* expression is elevated in embryonic telencephalic progenitor domains of mouse brain development.** Embryonic day (E)14.5 sagittal sections acquired from Genepaint showing mRNA expression of the *Odc1* transcript in mice. While expression is enriched in different brain regions (darker signal), the progenitor domains of the telencephalon have consistent elevated mRNA levels in both medial and lateral tissue sections (a, b). Arrows denote different progenitor domains that give rise to Glutamatergic neurons and cortical glia (Red) and GABAergic neurons (Blue).

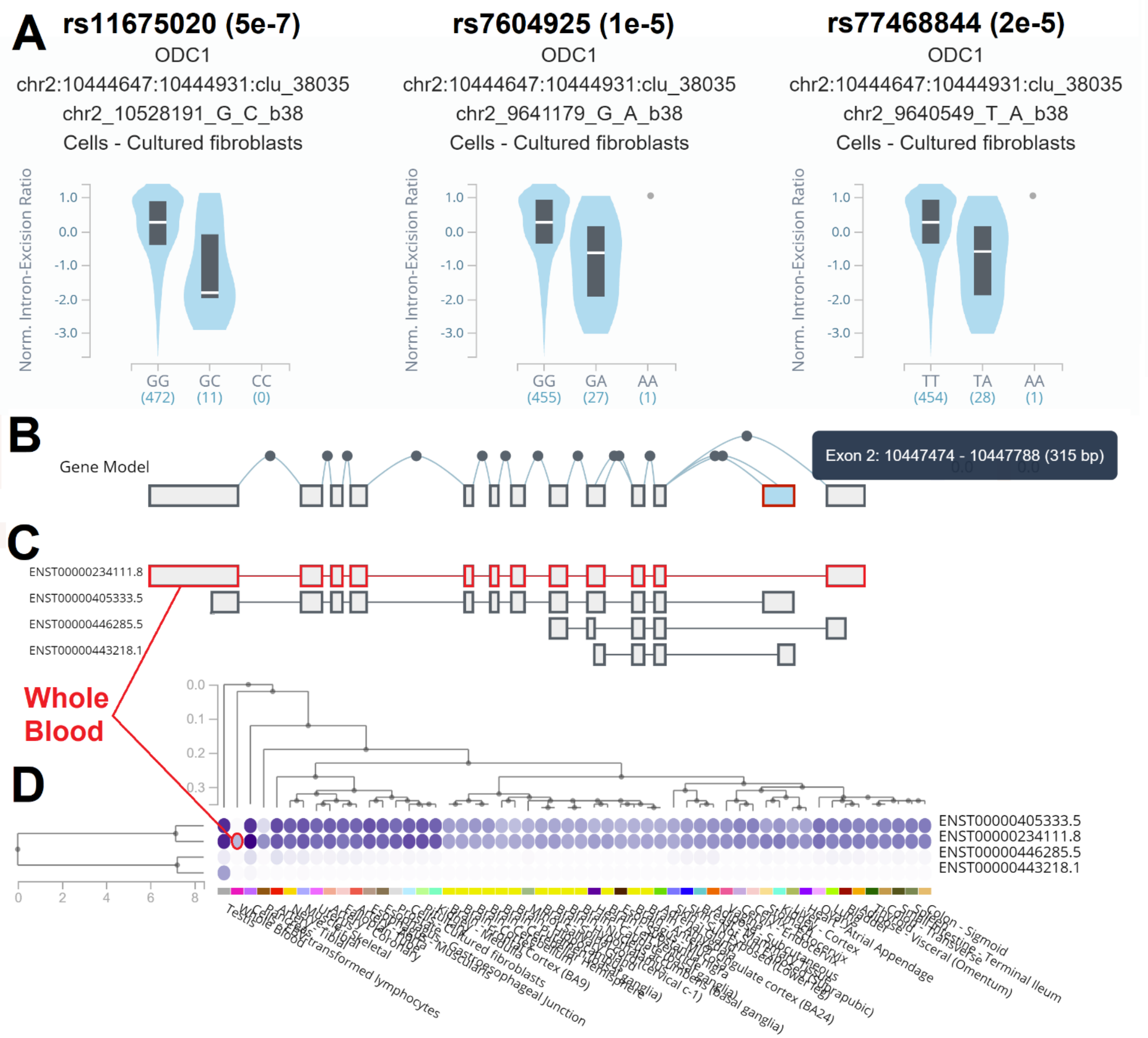


**Figure S13 Splicing QTL for *ODC1*. A)** Violin plots for three SNPs (with p-value) for exon1/exon2 to exon 3 splicing QTL of *ODC1* from GTEx v8. **B)** Splicing site changes with the red and blue exon changed in panel A. **C)** Known isoforms for *ODC1* with the isoform of panel A shown in red. **D)** Expression heatmap for multiple tissues (x-axis) of four isoforms of *ODC1* with whole blood utilizing the isoform of panel A sQTL.

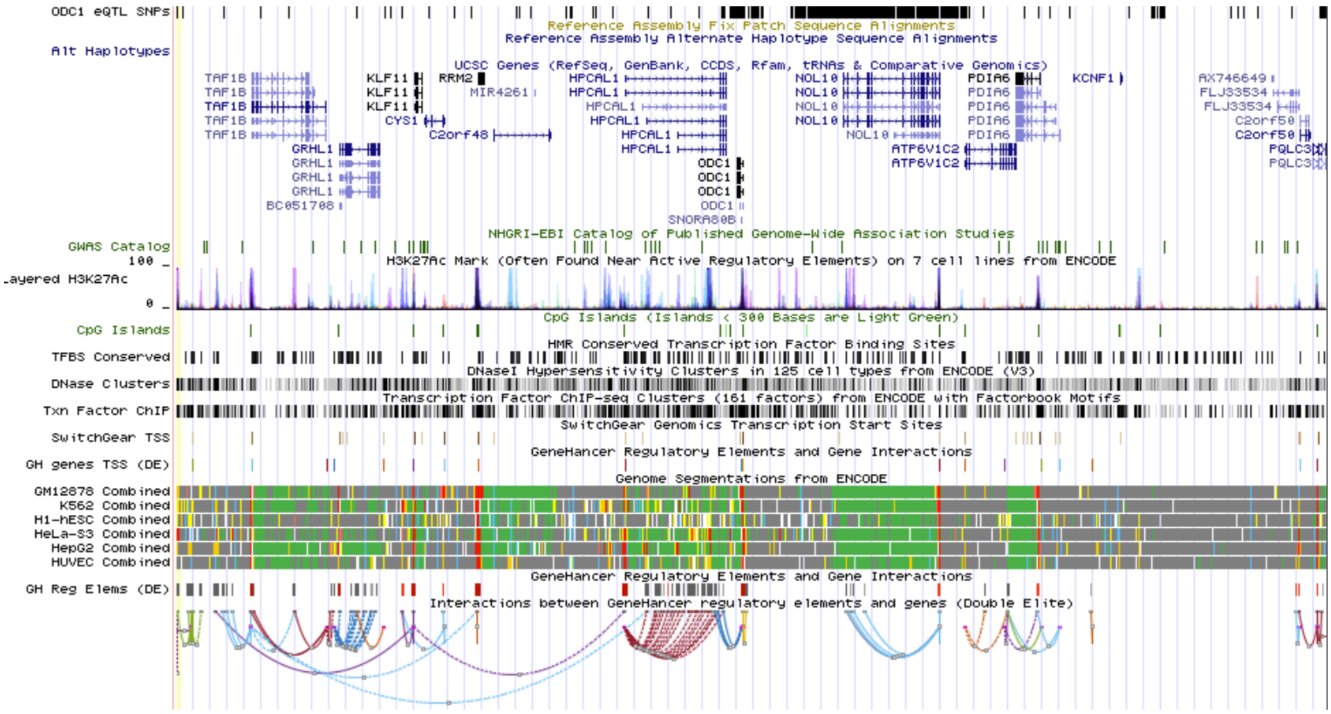


**Figure S14 Genomic regions for eQTLs connected to *ODC1* levels.** Shown on the top are all of the eQTL LD SNPs. This is followed by genome tracts for genes, GWAS linked SNPs, H2K27Ac data, CpG islands, conserved transcription factor binding sites, DNaseI hypersensitivity, ENCODE ChIP-Seq, SwitchGear start sites, GeneHancer locations, genome annotated segmentation for ENCODE cell lines, and GeneHancer looping data. These tracts were extracted for integrative analysis of each *ODC1* associated eQTL.

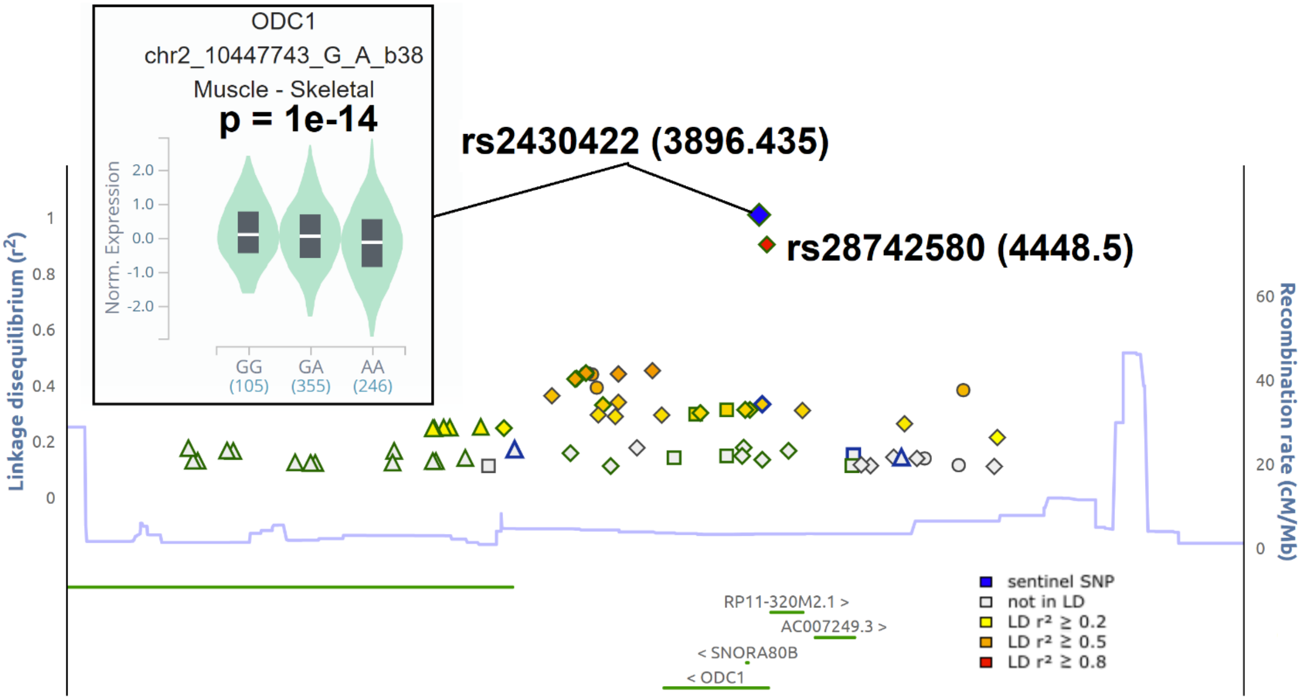


**Figure S15 rs2430422 Linkage disequilibrium SNPs**. rs2430422 is marked in blue with all LD SNPs with >0.8R2 SNP (rs28742580) in red. Next to each SNP is the annotated score and called out is shown the violin plot for eQTLs of rs2430422 for skeletal muscle. Shown on the bottom is the map of genes.

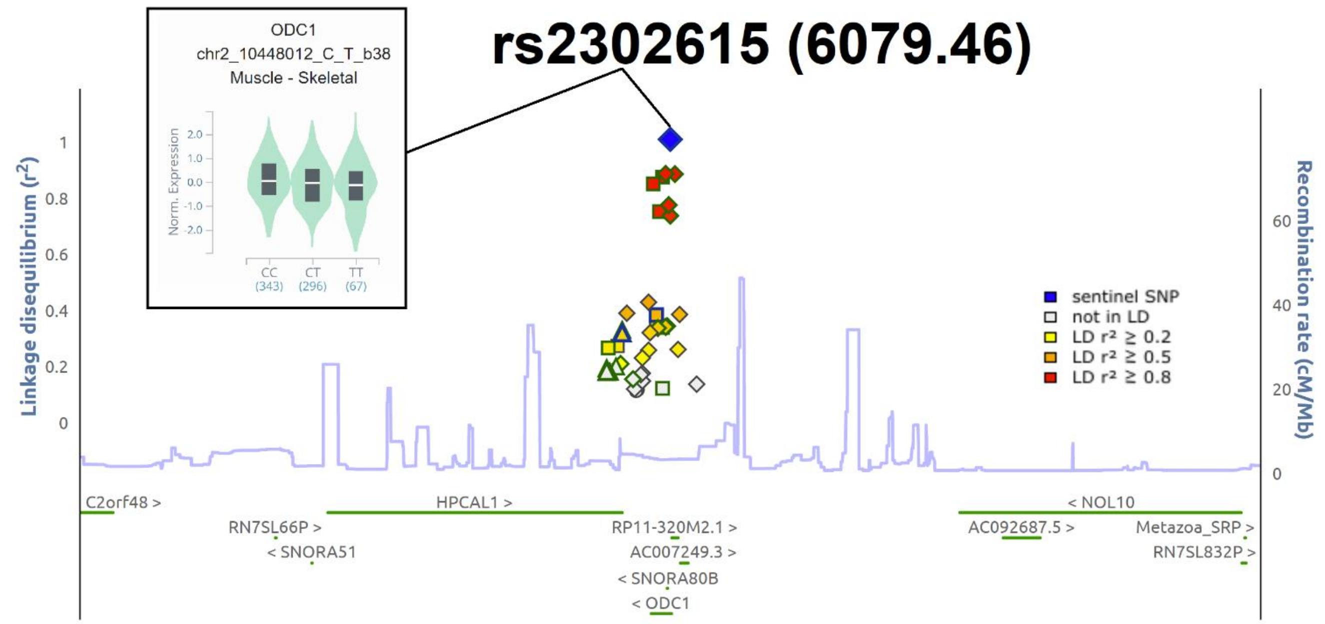


**Figure S16 rs2302615 Linkage disequilibrium SNPs.** rs2302615 is marked in blue with all LD SNPs with >0.8R2 SNP in red. Next to rs2302615 is the annotated score and called out is shown the violin plot for eQTLs of rs2302615 for skeletal muscle. Shown on the bottom is the map of genes.

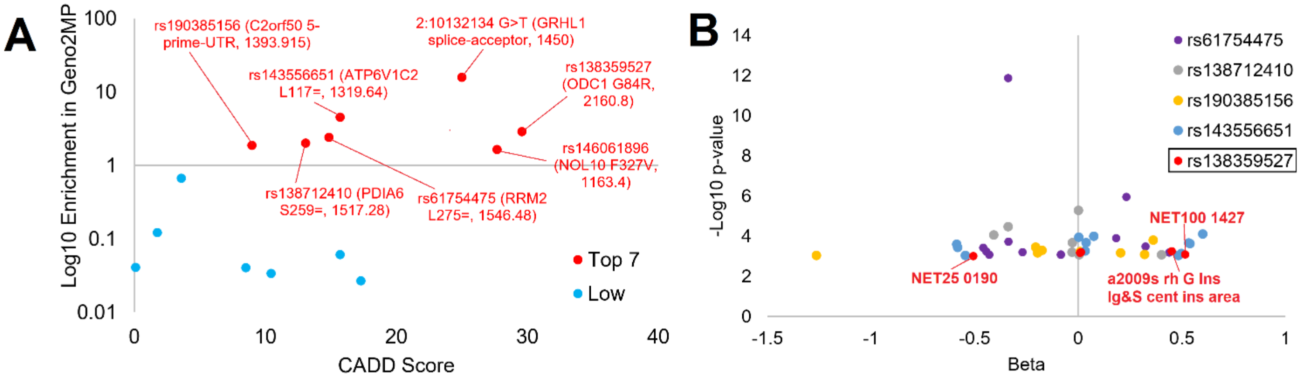


**Figure S17 Enriched SNPs with high impact from Geno2MP in *ODC1* associated genomic region**. **A)** The seven highest ranked Geno2MP SNPs (red) within the *ODC1* eQTL linked genomic region showing CADD score (x-axis) and the Log10 enrichment (y-axis), observed frequency within Geno2MP vs expected frequency based on gnomAD. Each SNP is labeled with rsID, gene, variant annotation, and combined score. Shown in blue are seven SNPs of low enrichment with at least 10 occurrences within Geno2MP. **B)** PheWAS returns for Beta (x-axis) and -log10 p-value (y-axis) for top SNPs in panel A. Details of traits are shown in Table S9. Neurological traits connected to rs138359527 are labeled in red.
